# Pathway Relevance Ranking for Tumor Samples through Network-Based Data Integration

**DOI:** 10.1371/journal.pone.0133503

**Published:** 2015-07-28

**Authors:** Lieven P. C. Verbeke, Jimmy Van den Eynden, Ana Carolina Fierro, Piet Demeester, Jan Fostier, Kathleen Marchal

**Affiliations:** 1 Department of Information Technology, Ghent University—iMinds, Ghent, Belgium; 2 Department of Plant Biotechnology and Bioinformatics, Ghent University, Ghent, Belgium; Roswell Park Cancer Institute, UNITED STATES

## Abstract

The study of cancer, a highly heterogeneous disease with different causes and clinical outcomes, requires a multi-angle approach and the collection of large multi-omics datasets that, ideally, should be analyzed simultaneously. We present a new pathway relevance ranking method that is able to prioritize pathways according to the information contained in any combination of tumor related *omics* datasets. Key to the method is the conversion of all available data into a single comprehensive network representation containing not only genes but also individual patient samples. Additionally, all data are linked through a network of previously identified molecular interactions. We demonstrate the performance of the new method by applying it to breast and ovarian cancer datasets from The Cancer Genome Atlas. By integrating gene expression, copy number, mutation and methylation data, the method’s potential to identify key pathways involved in breast cancer development shared by different molecular subtypes is illustrated. Interestingly, certain pathways were ranked equally important for different subtypes, even when the underlying (epi)-genetic disturbances were diverse. Next to prioritizing universally high-scoring pathways, the pathway ranking method was able to identify subtype-specific pathways. Often the score of a pathway could not be motivated by a single mutation, copy number or methylation alteration, but rather by a combination of genetic and epi-genetic disturbances, stressing the need for a network-based data integration approach. The analysis of ovarian tumors, as a function of survival-based subtypes, demonstrated the method’s ability to correctly identify key pathways, irrespective of tumor subtype. A differential analysis of survival-based subtypes revealed several pathways with higher importance for the bad-outcome patient group than for the good-outcome patient group. Many of the pathways exhibiting higher importance for the bad-outcome patient group could be related to ovarian tumor proliferation and survival.

## Introduction

Uncovering the molecular mechanisms that give tumor cells their growth advantage remains a fundamental challenge in cancer research. This task is non-trivial because cancer is a complex disease: a tumor’s growth advantage often is not caused by genetic alterations of a single type but rather by a combination of defects of different types. Consequently, the study of tumor development and progression requires the availability of different types of data. Each data type can capture a different aspect of the tumor’s deviating (epi-) genetic state and metabolism. Because of the diverse causes of cancer, the success of applying any tumor analysis method is uncertain if it operates only on part of the available data.

Furthermore, when one is concerned with revealing the mechanism of action underpinning the tumor’s growth advantage, a network- or pathway-based approach is crucial. Because of the clonal nature of tumor cells, true oncogenic alterations (contrary to e.g. passenger mutations that don’t contribute to the tumors fitness) are sparse. Patients with the same disease phenotype often will not share any somatic mutations occurring in the same pathway [1–5]. This so-called mutual exclusivity of somatic mutations, a concept that can be extended to other data types, renders the statistical task of identifying true genomic causes of cancer challenging and motivates ‘pathway driven’ analysis [1,4,6,7]. Such an analysis is no longer gene-centric, but exploits the fact that interacting genes constitute pathways, connecting upstream genetic disturbances (causes) with downstream effects. For tumors to exhibit a similar molecular or clinical phenotype, it is not required that they share the same disturbances in the same individual genes. Instead the same pathways will be impacted by possibly many combinations of (epi-)genetic alterations. These pathways can be identified by pooling the information present in different independently evolved tumors.

Tackling tumor analysis problems consequently not only requires multi-omics datasets and large patient cohorts, but also largely depends on the availability of an analysis framework that can integrate data of different types in a biologically relevant way [1,8–10]. We present a network-based data integration strategy that uses sets of genes (pathways) as the unit of analysis rather than individual genes. In contrast to existing tumor analysis methods, addressing either patient subtyping [1,11], driver gene prioritization [2–4,12,13], pathway impact assessment [6,7,14] or interaction network delineation [1,5,15], our method takes as input any combination of data (e.g., gene expression, mutation, copy number and methylation data). It allows for the ranking of pathways according to their relevance for a set of patients. To achieve this, all available data are cast into a unique network model. The model not only contains genes, but also the individual patients and prior knowledge in the form of a network of known gene interactions derived from public databases. Including patient samples as entities in the integrated network allows for quantifying the relevance of groups of genes for groups of patient samples using an intuitive measure of connectedness in this network representation. The gene interactions added as prior knowledge introduce mechanistic relations between individual genes in the network and will help relating diverse upstream genetic disturbances in the same pathway to the same molecular (downstream) phenotype, e.g. over– or under-expression of another set of genes.

Our contribution consists of a new method for pathway impact assessment. The method ranks a set of predetermined pathways according to their relevance for a given set of patient samples, and allows for the integration of any data type that can be cast into a binary relation between a gene and a sample or patient. It is an intuitive alternative to other methods incorporating pathway topology in their analysis like Signaling Impact Analysis [14] operating exclusively on gene expression data, and PARADIGM [6] operating on gene expression and copy number data. Both methods do not support the inclusion of mutation data. PARADIGM SHIFT [7], an extension of PARADIGM, does handle mutation data, but it is targeted specifically at the prediction of the nature of mutations by analyzing their downstream effects. We applied our method to two datasets from The Cancer Genome Atlas (TCGA), breast cancer [9] and ovarian cancer [16]. The method’s ability to rank pathways that are relevant to homogeneous groups of patients is demonstrated using the simultaneous analysis of mutation, mRNA expression, copy number and methylation data sets.

## Materials and Methods

### Pathway relevance ranking using network-based data integration

**Fig 1:**
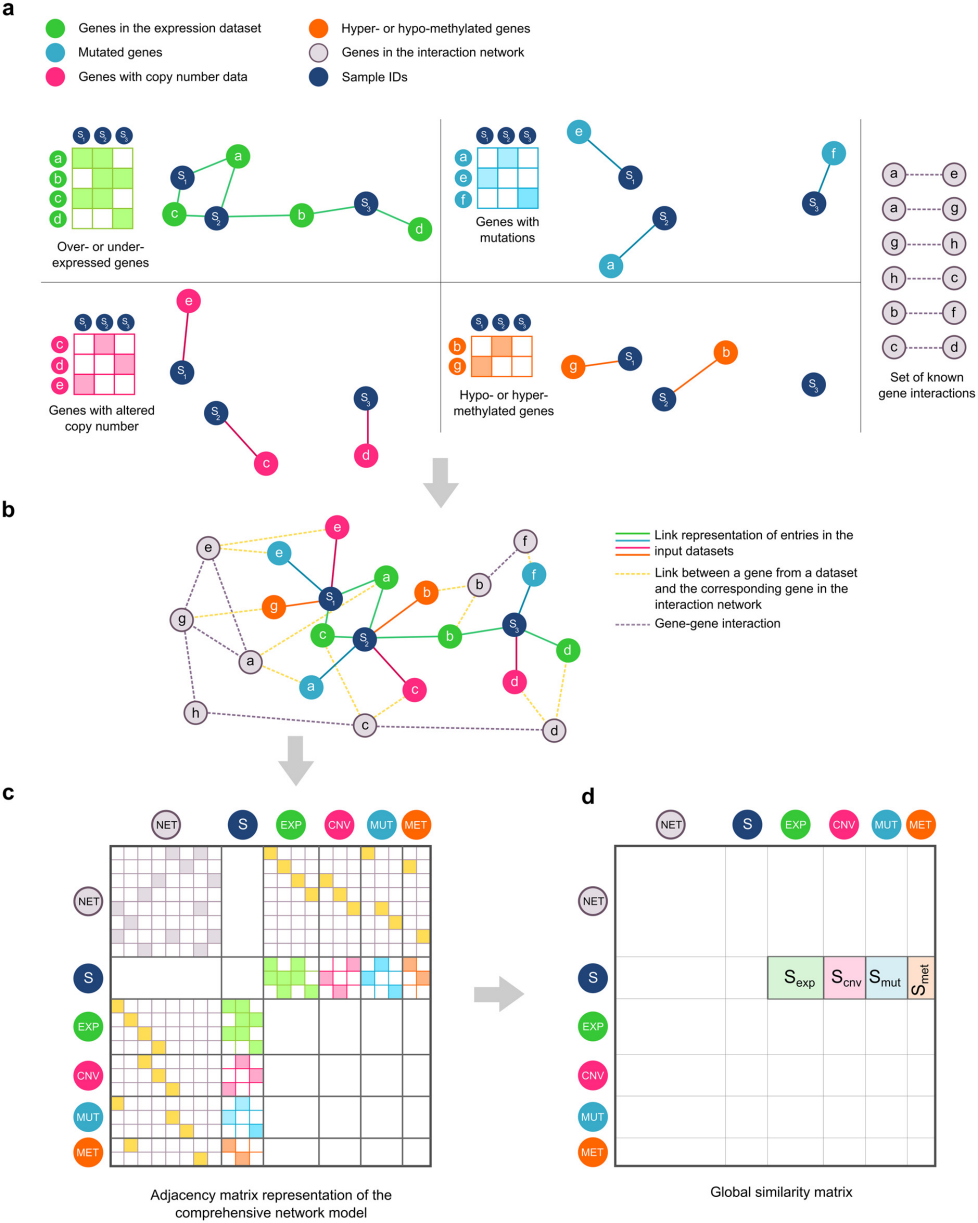
Global network construction. **(a)** Conversion of binary data to a network representation. All continuous data are mapped to a binary representation with ‘1’ (colored squares) corresponding to a gene with a value deviating from normal for a particular sample. Each ‘1’ in the binary datasets is converted to an undirected link (solid line) between a gene node and a sample node. Prior knowledge, derived from public gene interaction repositories, is available in the form of undirected links (dashed grey line) between genes. Characters a-g correspond to gene IDs, S_1_-S_3_ represent sample IDs. **(b)** Construction of the global network. The network representations of the binary datasets and the prior knowledge network are merged to constitute a single comprehensive network representation. Gene nodes originating from the input datasets are connected to the corresponding gene in the prior knowledge interaction network (dashed yellow lines). **(c)** The resulting adjacency matrix representation of the undirected global network. For clarity, individual gene and sample identifiers are omitted. NET (grey) = genes from the prior knowledge interaction network, S (dark blue) = samples, EXP (green) = genes from the gene expression dataset, CNV (pink) = genes from the copy number dataset, MUT (light blue) = mutated genes, MET (orange) = methylated genes. **(d)** The similarity matrix derived from the adjacency matrix, indicating the parts of the similarity matrix that are relevant for the pathway ranking task.

**Fig 2:**
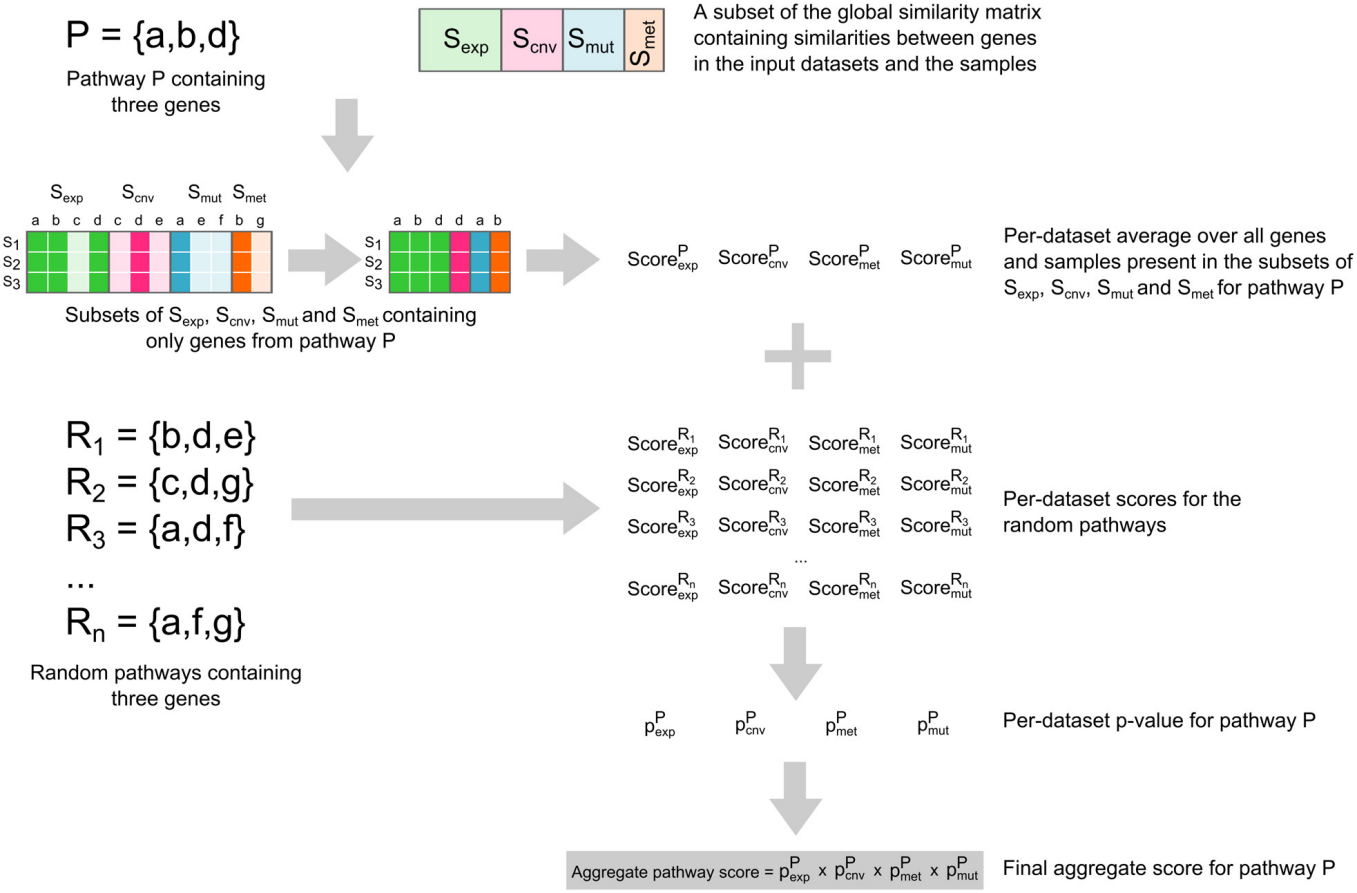
Pathway relevance scoring. Given a subset of the global similarity matrix (S_exp_ S_cnv_, S_mut_, S_met_, see Fig 1) and a set of genes (a,b,d) constituting a pathway *P*, a score for each input dataset is calculated by first removing genes from S_exp_ S_cnv_, S_mut_, S_met_ that do not belong to the pathway and then taking the average of all remaining values in S_exp_ S_cnv_, S_mut_, S_met_. This process is repeated for *n* randomly generated gene sets (with the same number of genes as the pathway *P*) yielding *n* scores for each input dataset. The random pathway scores are used to calculate a *p*-value for obtaining the pathway scores purely by chance. The resulting *p*-values are multiplied, resulting in a single aggregated pathway score.

We present an unsupervised analysis method that combines different data types with prior knowledge resulting in a comprehensive network representation (see below). Key to the method is the representation of all available data in a single network (referred to as the *global* network). This network contains all entities under study (patient samples, differentially expressed genes, genes containing mutations, …) and their mutual relations derived from either the data themselves or from prior knowledge. Using this global network representation, a similarity measure that expresses the degree to which network entities are related to each other can be calculated. Those similarities are subsequently used to assess the relevance of different pathways for a set of patient samples. The pathways, or more general, the sets of genes under study, need to be specified in advance. The proposed method ranks the predefined pathways according to their relevance for a set of samples. An overview of the method is given in Figs 1 and 2. A reference implementation is available for download from http://bioinformatics.intec.ugent.be/pathwayranking.

#### Global network construction

The construction of the global network is demonstrated using gene expression, mutation, methylation, and copy number data. Input datasets are required to contain data for all samples, but can contain data different genes. Datasets can be omitted, and any data that can be converted into a binary form can be added. Indeed all continuous data are necessarily made binary (see Data section). A ‘1’ corresponds to either over- or under-expression (i.e., differential expression), copy number amplification or loss, mutation or hyper- or hypo-methylation. Conversely, a ‘0’ corresponds to normal expression, normal copy number, the absence of mutations or a normal methylation pattern. Treating over-expression of a gene identically to under-expression (and analogously, treating copy number amplification and loss, and hyper- and hypo-methylation in the same way) may seem as an oversimplification. In practice, when studying homogeneous groups of samples (e.g., belonging to an identical subtype), it is less likely that some samples in this homogeneous group would exhibit over-expression and others under-expression of the same gene. A ‘1’ in a binary dataset merely reflects an abnormal state of a gene for a particular sample, and we assume that this state of abnormality will be similar for samples exhibiting the same disease phenotype. Note that because expression data of tumor samples are analyzed relative to expression data of normal samples (see Data section), differential expression in this context does not necessarily correspond to absolute high or absolute low expression.

To construct the global network, first each binary input dataset is represented as an individual network (Fig 1a). This is achieved by converting each ‘1’ in the binary data to an undirected link connecting a sample node (labelled *S*_*1-3*_ in Fig 1) with a gene node (labelled *a-g*). For example, if gene *a* is over-expressed in sample *S*_*1*_, a link between node *S*_*1*_ and node *a* is created. As a result, four separate networks (one for each input dataset) are created. Next, the individual networks are merged (Fig 1b). In the merging process, sample nodes are joined (e.g., the resulting global network only contains one sample node S_1_) but gene nodes are not. Gene nodes represent an abnormal state for which the interpretation is different for each input dataset. A gene can be differentially expressed, mutated, etc. and merging genes nodes would discard this information. Consequently, the network will contain multiple gene nodes with the same gene identifier. For example, in Fig 1b, gene *a* is present multiple times, once as a differentially expressed gene, and once as a mutated gene.

Additionally, in order to connect heterogeneous and potentially sparse genetic aberrations with their downstream effects on gene expression, prior information in the form of known gene interactions is incorporated. The prior knowledge interaction network will ensure that a pathway is not treated as an isolated group of genes. Instead, the genes in pathway are analyzed not only relative to each other, but also relative to other pathways that may or may not overlap with the pathway under study. New nodes representing genes involved in known gene interactions are created. These ‘interaction nodes’ are connected with undirected links whenever an interaction between these nodes exists (Fig 1a and 1b). Then, the genes derived from the input datasets, representing the different abnormal states, are connected to the interaction node with the same identifier. For example, the two *a* nodes in Fig 1b, representing a differentially expressed and mutated state of gene *a*, are both connected to a single interaction node *a*.

Note that the newly constructed global network no longer corresponds to a physical or functional gene interaction network. Instead, the network representation is a convenient means for integrating different types of data, including prior knowledge. This is possible because all links in the network can be identically interpreted, i.e. as a qualitative “is relevant to” relation. Because the network is treated as undirected, the resulting adjacency matrix representation is symmetric. As illustrated in Fig 1c, several parts of the adjacency matrix remain empty.

#### Network-based similarity calculation

The adjacency matrix representation of the global network (Fig 1c) is used to derive the similarity (a quantitative measure of relevance or importance) between samples and the gene nodes representing the abnormal states of the genes in the different datasets. The similarities are summarized in the global similarity matrix (Fig 1d). Similarity measures based on shortest path calculations would be an intuitive choice, but these measures have been shown to underperform, especially when data are qualitative, incomplete or of unknown reliability [17,18]. Because of their good performance [18–21], we used kernels calculated on graph nodes. A great multitude of kernels on graph nodes exist (see [22] for a comprehensive overview). Preliminary experiments suggested that the Laplacian Exponential Diffusion (LED) kernel yields stable results. It is calculated on the weighted Laplacian matrix *L* as follows [22]:

L=D−A

$$D\left(i,i\right)=\sum_{j=1}^{n}A\left(i,j\right)$$

$$K_{LED}=\text{exp}\left(-\alpha L\right)$$

Here *n* is the number of entities in the global network, *D* is the diagonal degree matrix and *A(i*,*j)* represents entry *j* on row *i* of the global network’s adjacency matrix *A*. *K*_*LED*_ *(i*,*j)* contains, at time *t = α*, the quantity found in node *i* when a unit quantity starts diffusing from node *j* at *t = 0*. The *exp*-operator indicates the matrix exponential. Calculating K_LED_ results in a similarity matrix with the same size as the original global adjacency matrix. Before continuing with the analysis, additional normalization of the similarity matrix is performed by exploiting the kernel properties of a kernel matrix *K* with elements *k*_*ij*_:

$$K_{N}\left(i,j\right)=\frac{k_{ij}}{\sqrt{k_{ii}\cdot k_{jj}}}$$

#### Pathway relevance ranking

When assessing pathway importance, the goal is to identify which pathways—containing active or inactive genes, mutated genes, genes with altered copy number or hyper- or hypo-methylated genes—are more relevant for a group of samples than others. Pathway relevance will typically correspond to abnormal behavior like activation or silencing and/or to the presence of genomic alterations. The pathways of interest should be available as sets of genes. Providing topology information for the pathways of interest is unnecessary, as the relations between genes are provided by the prior knowledge interaction network.

The pathway ranking problem can be addressed by focusing on the submatrices *S*_*exp*_, *S*_*mut*_, *S*_*cnv*_ and *S*_*met*_ derived from the global similarity matrix (Figs 1d and 2). These submatrices represent the similarities between the considered patient samples and genes measured in respectively the expression, mutation, copy number and methylation datasets. A high value for a sample-gene similarity indicates greater importance or relevance of a gene for that sample, and low values suggest that the gene is not important for the sample. Although each entry in these submatrices expresses a similarity between a sample and a gene present in one of the aforementioned datasets, the similarities are calculated using the entire global network. The similarity metric exploits the fact that the pathways under study are not isolated entities in the global network (note that the global network is more comprehensive than the prior knowledge interaction network). If the pathways were isolated entities, the average similarity per pathway would correspond to the average number of ‘ones’ in the pathway. Instead, pathways are interconnected through a number of mechanisms: (1) samples having ‘abnormal’ genes in different pathways or in different types of data will act as bridges between pathways and datasets, (2) pathways will typically intersect or overlap with each other and (3) the prior knowledge network also contains genes that are not present in the pathway compendium and that will act as bridges between pathways. The extra connections allow ‘abnormal’ genes from outside the pathway or from another datatype to contribute to the importance of a pathway for a particular datatype. As a result, it is possible for a mutated gene to be similar to a sample without actually being mutated in that particular sample. This can happen when the sample shares e.g. many differentially expressed genes with several other samples that do exhibit mutations in that gene.

The calculation of a single aggregate pathway score is illustrated in Fig 2. First, the matrices *S*_*exp*_, *S*_*mut*_, *S*_*cnv*_ and *S*_*met*_ are filtered so that they contain only genes that are present in the pathway under study. For example, gene *c* is not present in pathway *P* (containing genes *a*,*b* and *d*). Therefore, the third column of *S*_*exp*_, corresponding to gene *c*, is removed. Next, for each filtered submatrix separately, the average (over all samples and genes) of the remaining similarity values in *S*_*exp*_, *S*_*mut*_, *S*_*cnv*_ and *S*_*met*_ is calculated, resulting in a single score per input dataset (${Score}_{exp}^{P},\;{Score}_{cnv}^{P},\;{Score}_{mut}^{P}$ and ${Score}_{met}^{P}$).

Because a pathway’s score will be influenced by its size, and in order to eliminate random effects, the scores-per-dataset are converted to p-values that reflect the probability of observing (at least) the obtained score purely by chance. To achieve this, a large number of random gene sets are created (in this study, we used 10,000 permutations, see implementation details below). For each random set *R*_*i*_, a score is calculated for each input dataset (${Score}_{exp}^{R_{i}},\;{Score}_{cnv}^{R_{i}},\;{Score}_{mut}^{R_{i}}$ and ${Score}_{met}^{R_{i}}$) using the same approach as used for the pathway under study. The random score distribution obtained for each dataset then allows for the calculation of the probabilities $p_{exp}^{P},\;p_{cnv}^{P},\;p_{mut}^{P}$ and $p_{met}^{P}$. Multiplying these *p*-values results in a single aggregate pathway score that can be used to compare pathways.

The aggregate score should not be used as an absolute measure of relevance as it does not correspond to a true joint-probability. The constituting probabilities calculated for each input dataset will very likely be correlated. Consequently, the true joint probability of observing these per-dataset similarity scores will likely be higher than the value obtained by multiplying the individual p-values. However, we can assume that the correlation between the scores obtained for each input dataset will be present for all pathways. Consequently, the aggregated score can still be used to rank pathways. It has the additional advantage that it can be broken down into four components that can be traced back to the input datasets.

Comparing the scores of pathways containing highly interconnected genes with scores obtained for random sets of, most likely, unconnected genes may appear counter-intuitive: the connectivity of the pathways might result in higher scores that could never be achieved by sets of unconnected genes. However, the p-value of both a pathway and a random set of genes is calculated using the average similarities obtained for individual genes. Those similarities in turn are a function of the abnormal states observed in the different samples (the ‘ones’ in the input datasets), and of the way the gene is connected to other relevant genes in the network. Importantly, a gene’s score is not determined by how well the gene connects to the other genes that are present in the gene set under study, be it a pathway or a random gene set. Furthermore, because all genes in the analysis are present in the prior knowledge network (genes that are not present in the prior knowledge network were filtered out), all genes have neighbors in the network. Consequently, it is perfectly possible for a gene of a random gene set to be very important, either because it contains a lot of abnormal ‘ones’, or because it lies in a network neighborhood containing other important genes that are not required to be present in the random gene set.

As an alternative to the proposed permutation strategy, one could also permute the gene labels of the input datasets, and each time measure the score for the pathway under study. However, for each permutation, the computationally very expensive calculation of the similarity matrix is required. As illustrated in S1 Fig, the probabilities obtained by the computationally expensive permutation of the dataset gene labels are highly correlated (ρ>0.99) with the probabilities obtained using the proposed method. Consequently the fast random gene set based permutation strategy was used for all subsequent analysis.

#### Data binarization

In order to integrate an omics dataset in the global network representation, it needs to be converted into a list of qualitative gene-sample links or relations. Each entry in each input dataset is converted into either ‘0’ or ‘1’, representing respectively the absence or the presence of a meaningful link between a sample an a gene in the global network. Although many binarization strategies are possible, the most trivial approach was adopted: a hard threshold was set to the (absolute) values in a dataset. The dataset-dependent threshold is chosen in such a way that the fraction of entries in each data matrix that is set to ‘1’ is as a close as possible to a predefined parameter. In order to find the per-dataset threshold, a naive iterative procedure is used. This procedure is applied to all but the mutation datasets. The latter are already binary and are included without further filtering.

### Data

#### Gene expression, mutation, methylation and copy number data

For breast cancer, mRNA, mutation and copy number data were downloaded from The Cancer Genome Atlas (TCGA, https://tcga-data.nci.nih.gov/tcga) in November 2012 (corresponding to the data from [9]), and methylation data were downloaded in October 2013. 463 patients had data points for all data types and were retained in the final analysis. The ovarian cancer datasets were downloaded from TCGA in April 2014, and contained mRNA, mutation, methylation and copy number data for 447 patients. All breast and ovarian cancer data used in this study are part of the TCGA open access data tier containing only de-identified and anonymized data. For breast cancer, we focused on patients with Her2, Basal, Luminal A and Luminal B tumors. Patients with Normal-like tumors were omitted from the analysis because of the low number of available samples. All gene identifiers were mapped to Entrez gene identifiers. Genes that could not be mapped were left out of the analysis.

To reduce the problem size, all datasets were filtered to contain only genes that are present in the prior knowledge interaction network (see below). For the mRNA datasets, this resulted in a final selection of 10100 and 9463 genes for respectively breast cancer and ovarian cancer. Expression data were centered on the median of the available normal samples, and made binary according to the procedure laid out in the Method section. Rather than corresponding to absolute high or absolute low expression values, the binary data will reflect whether a gene was differentially expressed, relative to normal tissue data. No variance scaling was applied as this would increase the impact of noise for genes with very low and constant normal expression.

The mutation data were preprocessed using MutSig [13]. In order to obtain a broader selection (with a possibly high number of false positives), we used unadjusted p-values with a cut-off of 0.05. For breast cancer, the final mutation dataset contained 465 mutated genes with Entrez identifiers. For the ovarian cancer dataset, an identical procedure was employed resulting in 327 genes.

Copy number data for breast cancer and ovarian cancer were processed in the same way. Significant copy number regions were identified with GISTIC2.0 [23] using the same parameter settings as in the TCGA breast cancer overview [9]. The final dataset contained 446 and 2806 genes for respectively breast cancer, and ovarian cancer. The copy number data were made binary by applying a threshold to the absolute value of the copy number variation, according to the binarization strategy described above.

The methylation data were downloaded from TCGA and filtered using the procedure and parameters described in the ovarian cancer study of the Cancer Genome Atlas Network [16]. Summarizing, hyper-and hypo-methylated genes are kept in the data set only if the following conditions are fulfilled (parameters are for hyper-methylated genes and correspond to respectively relaxed and strict settings in the filtering procedure, for hypo-methylated genes the procedure is adjusted in a trivial way). (1) In normal tissue, the gene should only slightly be methylated (<0.5, <0.4). (2) The 90^th^ percentile methylation level of tumor samples should be considerably higher (0.1, 0.3) than the average normal methylation level. (3) The average gene expression of normal samples should be considerably lower (2, 3 fold) than the average gene expression of the 10% highest methylation levels in tumor samples. (4) There should exist a negative spearman correlation (<-0.2, <-0.3) between the methylation level and the gene expression level. Applying this procedure resulted in 624 hyper-methylated and 270 hypo-methylated genes for breast cancer and 42 hyper-methylated and 173 hypo-methylated genes for ovarian cancer. All methylation data are transformed from the [0,1] range to the [-0.5,0.5] range by subtracting 0.5. The methylation data were made binary according to the same binarization strategy used for the other data types.

#### Network data

KEGG pathway data [24] were downloaded on November 11, 2014 using the KEGG REST-API functionality. 224 pathways (disease pathways were left out of the analysis) were merged to constitute a single network. Non-gene entities (groups and complexes) in the KEGG topology were expanded using dummy genes (with unique identifiers) that were connected to the individual genes constituting the group or complex. Interacting genes with such groups or complexes are then connected with the dummy genes rather than with the constituting genes. Because the goal is to capture and use patterns of regulation, transcription factors and their targets were explicitly added. Transcription factor—target interactions were obtained from http://encodenets.gersteinlab.org/ which is part of the ENCODE project [25]. Only interactions based on proximal TFBS data were included in the final network. The final network contained 12040 genes and 97482 interactions.

### Parameter tuning and implementation details

The presented procedure is controlled by two parameters: the Laplacian Exponential Diffusion *α* parameter, and the desired fraction of entries in each dataset that will be set to ‘ 1‘ in the data binarization process. The diffusion parameter *α* was set to 0.01 for all experiments (for both breast cancer and ovarian cancer), but comparative experiments revealed that the obtained pathway rankings are stable for values of *α* between 0.0001 and 0.05. The average number of relations between genes and patient samples was set to 0.1 for all datasets.

The method was implemented in Matlab, and run on a 16-core, 64bit CentOS 6.2 system with 128GB of memory. Source code and sample data is available from http://bioinformatics.intec.ugent.be/pathwayranking/. The calculation of the matrix exponential determines runtime and memory usage which are known to be *O(N* ^*3*^ *)* and *O(N* ^*2*^ *)* respectively, with *N* the number of entities in the global network. To reduce runtime and memory requirements, the global adjacency matrix was compressed by merging the mRNA expression dataset with the prior knowledge network, resulting in a substantial reduction of problem size. The problem size *N* was further reduced by filtering out all genes that were not present in the prior knowledge network. The duration of the pathway ranking procedure is a linear function of the number of random permutations which is in turn depending on the desired minimum obtainable p-value. If a minimum p-value of 0.0001 is needed, scores for 10,000 permutations need to be calculated. Calculating scores for permutations is a problem that is so-called *embarrassingly parallel* (scores for individual permutations are independent of each other) indicating that the procedure is easily sped up in a multi-core environment. On the system used, the processing of a typical analysis run (with 10,000 permutations) was finished in less than 40 minutes.

## Results and Discussion

### Pathway importance ranking for breast cancer subtypes

**Fig 3:**
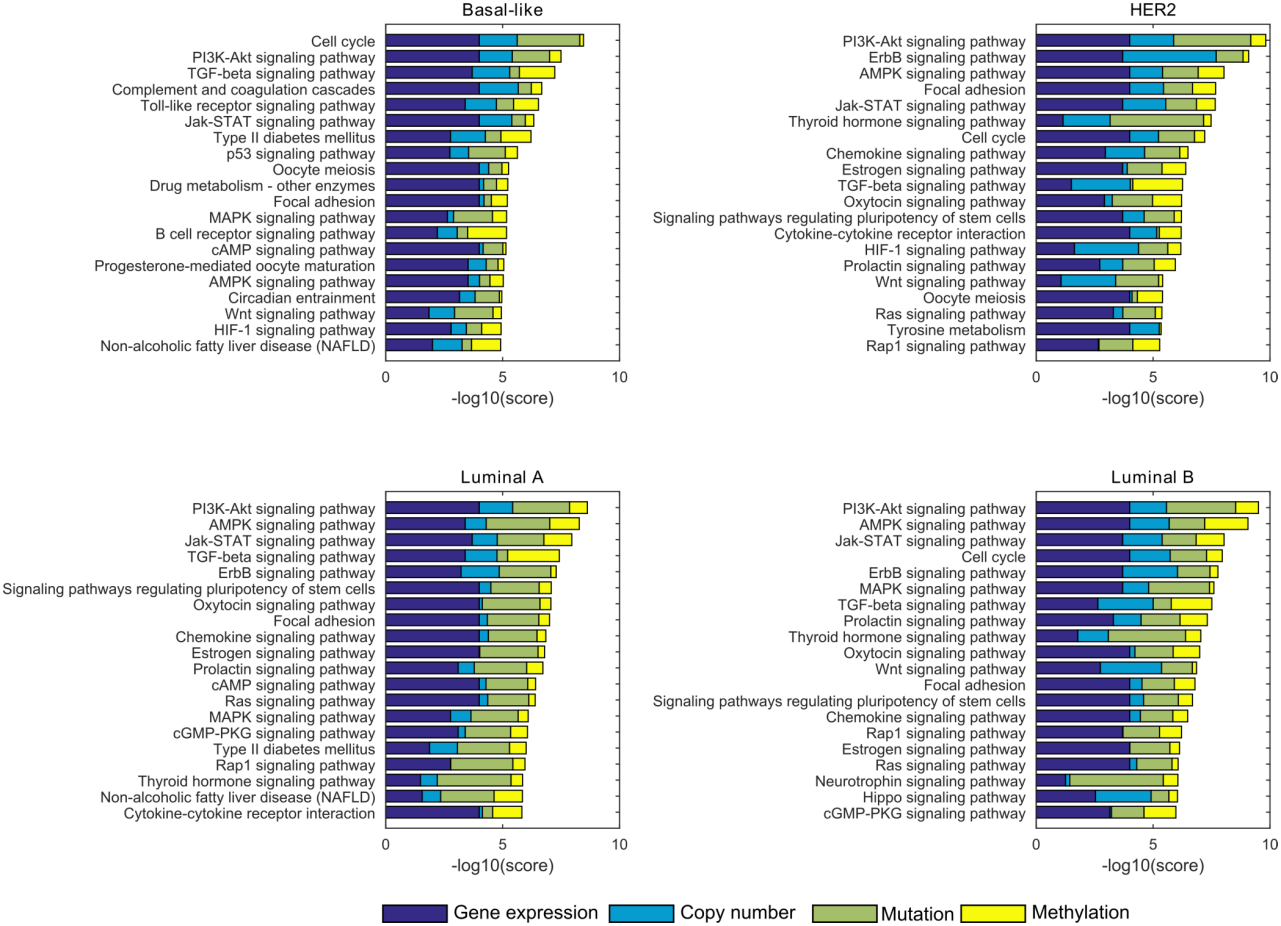
The 20 highest ranking pathways for each of the four breast cancer subtypes. The aggregate score assigned to each pathway can be decomposed into 4 probabilistic components. The contribution of each component to the total score is indicated in a different color bar: mRNA expression (dark blue), copy number (light blue), mutation (green) and methylation (yellow).

The importance of 224 non-disease KEGG pathways was assessed for each of the breast cancer subtypes by calculating a relevance score per pathway for each breast cancer PAM50 subtype (Basal-like, HER2, Luminal A, Luminal B) (Fig 3). Each relevance score is an aggregate of four components: mRNA expression, copy number, mutation and methylation. The application to breast cancer tumors serves as a validation of the newly developed method. The molecular PAM50 breast cancer subtypes have been extensively characterized [9], and the ranking procedure should recapitulate to a large extent what is known about these subtypes.

In general, the gene expression score component (or sub-score) is larger than the other components. This is to be expected, because the sub-scores reflect the probability of observing a particular expression, mutation, copy number or methylation pattern purely by chance. If a pathway is truly relevant for a subtype, we expect (1) that this is reflected by differential expression of a set of genes in that pathway, and (2) that the expression pattern of genes in that pathway will be consistent for tumor samples within that subtype (the downstream effect of a genetic aberration on gene expression will be similar). Conversely, mutation, copy number and methylation sub-scores are expected to be lower than the expression sub-score, since (1) oncogenic aberrations are sparse and (2) pathway-disturbing alterations often are not consistent even when they result in an identical downstream gene expression pattern (see the Introduction section). Furthermore, the subtypes are determined using a gene expression-based classifier. This ensures similar gene expression values for at least the genes that are present in the classifier. The more consistent genes behave within a single subtype, the higher the similarities between the samples and the genes of a pathway will be and the less likely such similarities will be observed purely by chance.

#### Subtype-independent pathways

The (mitotic) *cell cycle* pathway is the highest scoring pathway for the Basal-like subtype, but it is also ranked high for the HER2 and the Luminal B subtypes. It is absent in the top 20 for the Luminal A subtype. Closer inspection of the components making up the aggregate score (Fig 3, S2 Fig) indicates that the score is highly determined by a substantial copy number (predominantly transcription factor MYC, cyclin CCND1 and the MDM2 oncogene) and mutation (tumor suppressor TP53) component. Mutations of TP53 are absent in Luminal A tumors, explaining the low score of this pathway for that subtype. The HER2 and the Luminal B subtype also exhibit a small methylation component that appears to be determined by the methylation status of the CCND1 and CCND2 cyclins, CDKN1C (a negative regulator of cell proliferation) and the chromatin binding MCM5 protein (S2 Fig).

The *PI3K-Akt signaling* pathway, responsible for cell survival and proliferation through regulation of the AKT protein kinase, scores uniformly high for all subtypes. Yet, even though the constituting score components are of a similar order of magnitude for all subtypes, the actual genetic disturbances are diverse (S3 Fig). For instance, all subtypes exhibit mutations in this pathway, but for the Basal-like tumors, this is limited to a single gene (TP53), whereas the HER2 subtype is characterized by a combination of mutations in TP53 and the PIK3CA kinase. The Luminal A and B subtypes are characterized by fewer mutations in TP53, complemented with a combination of mutations in, among others, PIK3CA, the AKT1 kinase, the PTEN phosphatase and KRAS, a kinase and well-known proto-oncogene. A similar pattern can be observed from the copy number data, where in Basal-like tumors, a uniform amplification pattern is observed for MYC and PIK3CA, whereas the other subtypes exhibit a combination of amplified MYC, CCND1, MDM2, IKBKB (a serine kinase), MCL1 (involved in apoptosis regulation) and RPS6KB1 (a protein kinase). Finally, the presence of the *Basal-like—HER2 –Luminal A—Luminal B* methylation gradient (S3 Fig) is reflected in the presence of a small methylation component that increases in size according to the same gradient.

Because the *Jak-STAT signaling* pathway, involved in cytokine and growth factor signaling, shares many genes with the *PI3K-Akt signaling* pathway (e.g. MYC, CCND1, MYC, AKT1, AKT2, PIK3CA, and the PIK3R1 kinase, see S4 Fig), it also scores uniformly high across all subtypes. It scores lower than the PI3K-Akt signaling pathway because TP53 is not a member of the pathway, nor many of the additional mutations (KRAS, PTEN) found in the PI3K-Akt signaling pathway. This results in a smaller mutation component in the aggregate score (Fig 1).

The *TGF-Beta signaling* pathway scores very high for the Basal-like and Luminal A subtypes, and intermediate (top 10) for the HER2 and Luminal B subtypes. It is involved in many aspects of development biology, and in the regulation of a wide spectrum of cellular functions, including proliferation and apoptosis [26]. Its role in breast tumor development is ambiguous as *TGF-Beta signaling* can both suppress and promote cancer progression [27]. Interestingly, its score is, for all subtypes, determined by a large methylation component. Indeed no other pathway has (averaged over the subtypes) so large a methylation sub-score, which is remarkable since only 6 genes of the pathway are present in the methylation dataset (S5 Fig). A possible explanation lies in the topology and size of this pathway. The *TGF-Beta signaling* pathway representation in the KEGG database is relatively small and linear. This makes it easy to connect upstream methylation events (almost all methylated genes in the pathway are found at the start of the signaling cascade) with downstream expression effects, resulting in large similarity scores for those genes. Together with the *TGF-Beta* signaling pathway, the presence of the *WNT signaling* pathway for all subtypes but Luminal A is notable. For the Luminal B subtype, the *Hippo signaling* pathway is found too. These three pathways act as large cross-talking modules [26] and it has been suggested previously that these pathways play a prominent role in (triple negative) breast cancer development [15].

The pathways described above were all ranked high for (almost) all subtypes. Functionally, these pathways take part in biological processes related to cell division, proliferation and survival. Their role in breast tumor development is well studied [9]. Interestingly, the underlying disturbances leading to the high scores can be divers. Because disturbances are connected through the *a-priori* network, the effect on the pathway score of a set of mutual exclusive mutations or copy number alterations occurring in that pathway can equal (and indeed exceed) the effect of a single consistent disturbance. This is clearly illustrated by the *PI3K-Akt signaling* pathway, where similar scores are obtained for the Basal-like subtype exhibiting only TP53 mutations and MYC amplifications, and the other subtypes where TP53 mutations and MYC amplifications are much sparser, but are complemented with other disturbances.

#### Subtype-specific pathways

**Fig 4:**
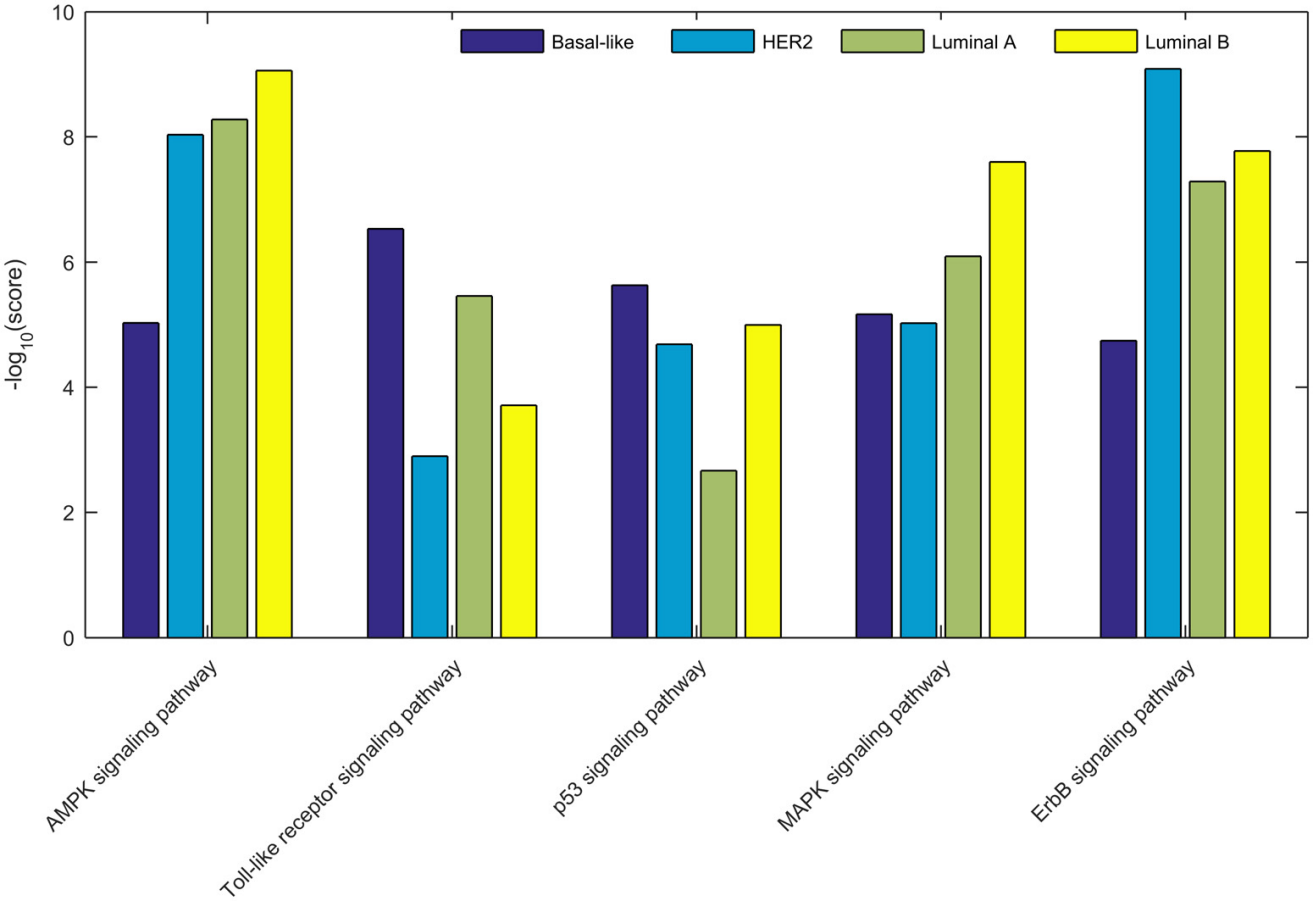
Pathway scores compared across breast cancer subtypes for a selection of pathways. Dark blue = Basal-like, light blue = HER2, green = Luminal A and yellow = Luminal B.

Because the analysis was performed separately for each subtype, the method is expected to identify not only pathways that are active or important for all subtypes, but also subtype-specific pathways. For a selection of differentially scoring pathways, Fig 4 displays a comparison of scores obtained for the different subtypes.

The scores for the *p53 signaling* pathway are highly determined by the mutation status of TP53 (S6 Fig) and consequently, the high score for the Basal-like subtype and the low score for the Luminal A subtype are to be expected. P53 signaling is intricately related with a cell’s response to, among others, DNA damage and activated oncogenes. Differential activation of this pathway in combination with hormone receptor status is an important factor determining breast cancer progression and outcome [28]. Even though Luminal B tumors too are only infrequently mutated in TP53, their score is much higher than for Luminal A tumors. A detailed investigation of the expression data revealed that Luminal B tumors express much more genes from the p53 signaling pathway than the Luminal A tumors do (S6 Fig), suggesting that the aberrant activity of this pathway is not necessarily caused by defects in the pathway itself.

The Toll-like receptor signaling pathway, suspected to be involved in the tumor’s evasion of the immune system [29], scores very high for the Basal-like subtype, intermediate for the Luminal A subtype, and low for the HER2 and Luminal B subtypes. The differences in scores for the subtypes appear to be caused by differences in the amplification and mutation status of PIK3CA, where again the lack of PIK3CA mutations in the Basal-like subtype are compensated by additional copy number amplification of PIK3CA, copy number amplifications of the MAP3K7 and RIPK1 kinases, copy number deletions in PIK3R1, and (slight) hypo-methylation of chemokine CCL5 and toll-like receptor TLR9 (S7 Fig).

The *ErbB signaling* pathway couples extra-cellular growth signals to intra-cellular signaling pathways that eventually control cell survival, proliferation and motility. It is involved in tumor development and progression, and many ErbB inhibitors are currently used therapeutically [30]. Its score is highly determined by the amplification state of the ERBB2 receptor tyrosine kinase and the mutation state of PIK3CA (S8 Fig). HER2 tumors exhibit an almost total amplification of ERBB2 that translates immediately to the overexpression of ERBB2 in this subtype. Luminal A tumors lack the amplification of ERBB2 but are, like HER2 tumors, frequently mutated in PIK3CA. Luminal B tumors are infrequently mutated in PIK3CA and exhibit infrequent ERBB2 amplification, whereas in Basal-like tumors PIK3CA is not mutated, nor is ERBB2 amplified.

The *MAPK-signaling* pathway is one of the few pathways (together with the AMPK pathway, see below) that scores highest for the Luminal B subtype. The ample evidence that aberrant MAPK signaling promotes tumor cell proliferation, survival and metastasis, makes this pathway an interesting inhibitory drug target [31]. MAP3K1, which is known for its critical function in cell fate decisions, is one of the central genes of the pathway and is crucial in connecting upstream and downstream genes [32]. Consequently, the observed differences in scores between the subtypes are expected to depend on its mutation status. Interestingly, although MAP3K1 is more frequently mutated in Luminal A tumors (S9 Fig), the pathway score is higher for the Luminal B tumors, and the mutation sub-score for Luminal B tumors is higher than for Luminal A tumors (Fig 3). This can be due to the fact that, like for the *p53 signaling* pathway, the Luminal B tumors express more genes from this pathway and several genes are (slightly) more methylated (S9 Fig). The more genes that are differentially expressed or methylated, the more connections exist (through the global network) between tumor samples and mutated genes and the higher the similarity scores between the tumor samples and the mutated genes will be. Conversely, the omnipresent mutations of TP53 in the Basal-like and HER2 subtypes do not result in a particular higher score for these subtypes, since the topology of the pathway is such that the connection between TP53 and the differentially expressed and methylated genes is less straightforward [24].

Like the *MAPK signaling* pathway, the *AMPK signaling* pathway, a sensor of cellular energy status [24], scores very high for the Luminal B tumors, intermediate for Luminal A and HER2 tumors, and low for the Basal-like subtype. Here the pathway score can be explained (S10 Fig) by the inter-subtype differences in PIK3CA mutations (absent in Basal-like tumors, very frequent in Luminal A), copy number amplification of CCND1 and RPS6KB1 (unimportant in Basal-like and Luminal A, frequent in HER2 and Luminal B) and copy number loss of PIK3R1 (Basal-like).

The analysis above indicates that the presented method is able to prioritize subtype-specific pathway importance with the high score for the *ErbB signaling* pathway for the HER2 subtype as a typical example. The results for the *p53* and *MAPK-signaling* pathway confirm that the integrated approach, where the analysis does not depend on a single gene nor a single type of data, is relevant and results in pathway scores that do not merely reflect mutation or copy number alteration frequencies.

#### Comparison with alternative approaches

To put the proposed method into perspective, we compared it to a naïve frequency-based approach. Instead of using, for each dataset, per-gene aggregated network-based similarity scores, we used the frequency of occurrence of ‘abnormal’ states (i.e. the number of times a gene was differentially expressed, mutated, … aggregated over all samples) as the individual gene score. Using the same permutation-based approach as used for the proposed method, a p-value for each pathway can be obtained for each dataset, and the p-values can be combined to result in a single frequency-based score per pathway. This naïve approach was applied to the Luminal A breast cancer subtype, corresponding to the largest group of patients in this study. The frequency-based pathway ranking was performed on the same (filtered) datasets that were used to obtain the network-based results. The results are displayed in S2 Table. In general, the two approaches agree: even though 224 pathways were evaluated, the 50 highest scoring pathways obtained with the proposed method can almost all be found among the 50 highest scoring pathways obtained with the frequency-based approach. However, the ordering of the pathways differs considerably between the two methods. Several relevant high-scoring pathways (see Result section above) obtained with the network-based method tend to be ranked lower by the frequency-based method, as is exemplified by the MAPK signaling pathway. This clinically important pathway is ranked 14^th^ by the network– based method, but ranked only 32^th^ by the frequency based approach. The pathway exhibits, more than any other pathway, a dispersed pattern of mutual exclusive mutations. The low mutation frequencies for individual genes result in a low rank when using only frequency as a relevance criterion.

Next, we compared our pathway relevance assessment results with those mentioned in the original PARADIGM publication [6]. Re-running PARADIGM with the same data used in our experimental setup, including mutation and methylation data, was not possible. We found that the top 15 pathways mentioned in the older PARADIGM study correspond to, or overlap highly with at least one pathway of our top-ranked pathways for each subtype (S1 Table). The only exception to this is the ‘*p75(NTR)-mediated signaling pathway’* ranked 6 out of 15 in the original PARADIGM study. This pathway maps to the *neurotrophin signaling* pathway in this study. In addition to the overlapping pathways, our approach also high-scored several other pathways (e.g. *TGF-Beta*, *WNT* and *Toll-like receptor* signaling). This is to be expected since firstly, in this study, additional datasets are used as input, and secondly, breast cancer subtypes are analyzed separately rather than collectively.

### Pathway importance ranking for ovarian cancer

For the ovarian cancer analysis, no molecular subtypes were used. Instead the 447 tumors were stratified based on survival data. We assume that strong differences in survival have a molecular foundation, although this may not always be the case. Three groups were identified: a group where patients were not alive 1000 days after the diagnosis (104 patients), a group that survived for at least 2000 days (47 patients), and an intermediate group (296 patients). We investigated whether the newly developed method is able to identify pathways that can help in explaining the different outcome between these groups of patients. Similar to the breast cancer analysis, the importance of 224 non-disease KEGG pathways was assessed for each of the survival-based subtypes. In what follows, we focus on the similarities and differences between the most extreme survival-based subtypes.

#### Important pathways common to all subtypes

**Fig 5:**
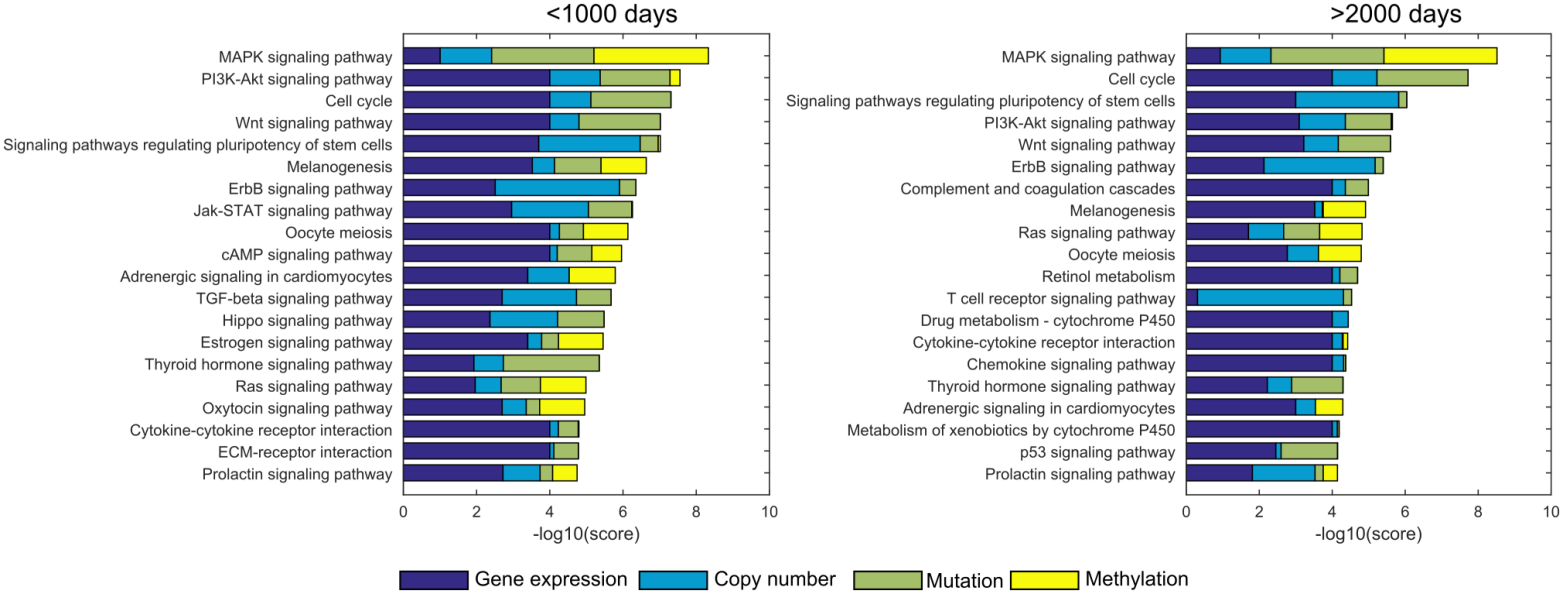
The 20 highest ranking pathways for the two most extreme ovarian cancer survival-based subtypes. The contribution of each component to the total score is indicated in a different color bar: mRNA expression (dark blue), copy number (light blue), mutation (green) and methylation (yellow).

Fig 5 displays the 20 highest scoring pathways for the two most extreme subtypes. From the figure, it is obvious that in general the patient group with the worst outcome has higher pathway scores than the group with the best outcome. Only the *MAPK signaling* and the *cell cycle* pathway exhibit similar aggregate scores. The fact that many different pathways are scored differentially between the subtypes suggests that the molecular explanation for the difference in survival will most likely not have a single cause.

In previous studies, as summarized in [16], a number of important deregulated pathways have been related to ovarian cancer: RB-signaling (cell cycle), RAS/PI3K signaling (PI3K-Akt signaling), FOXM1 (not present in the KEGG database, but overlapping with the *cell cycle* pathway) and Notch signaling. In this study, the highest scoring pathways are the *MAPK signaling* pathway, the *cell cycle* pathway, the *PI3K-Akt signaling* pathway and the *WNT signaling* pathway. The absence of the Notch signaling pathway in the top 20 of highest scoring pathways for either subtype is striking. A closer inspection of the actual data (S11 Fig) reveals that the Notch signaling pathway harbors few (epi-) genetic disturbances, both in terms of disturbances per gene as well as in terms of the number of disturbed genes. Additionally, as the KEGG representation of the Notch signaling pathway only contains 24 genes, it is possible that the pathway information is incomplete. However, a close cooperation between *MAPK signaling* and *Notch signaling* has been described in other tumors [33], and *MAPK signaling* is top-scored for all subtypes. The presence of the *WNT signaling* pathway, implicated previously in ovarian cancer tumorigenesis [34,35], is noteworthy. Its high score will likely be determined (S12 Fig) by TP53 mutations and copy number amplification in e.g. MYC, the NDK2 kinase and WNT5B (encoding for a signaling protein).

#### Differential subtype analysis

**Fig 6:**
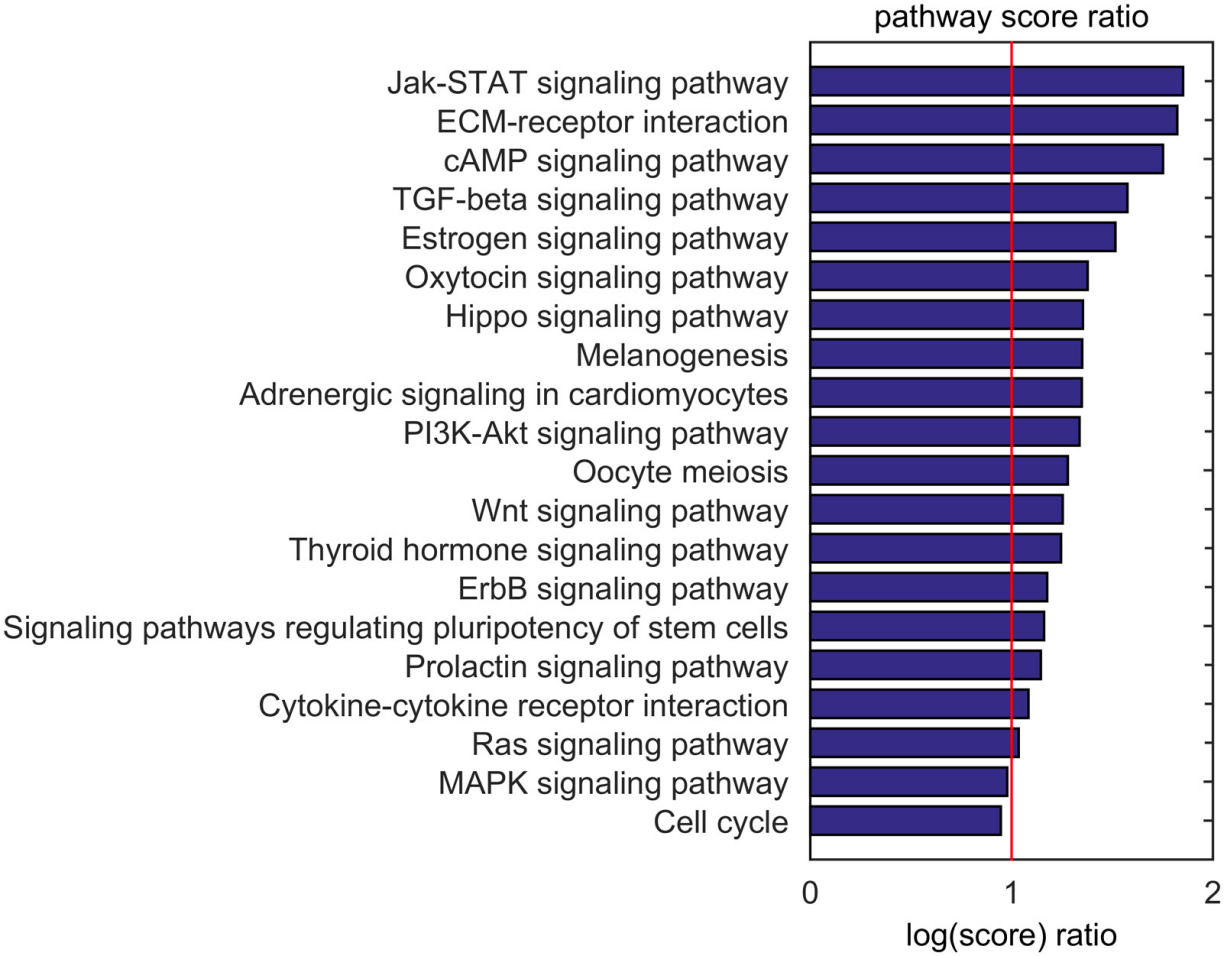
Ratio of bad-outcome pathway scores and the corresponding good-outcome scores. A ratio of ‘1’ indicates that the pathway scores equally high for patients in the bad-outcome group and patients in the good-outcome group. Values larger than 1 indicate higher pathway importance / activity for the bad-outcome group. Pathways shown are limited to the top-20 highest scoring pathways in the bad-outcome group.

More than in the pathways that score uniformly high across subtypes, we are interested in pathways that score differently for the survival-based patient groups. To achieve that, we focused on the 20 highest scoring pathways for the bad-outcome group. For each of these pathways, we calculated the ratio of the score for the bad-outcome group and the corresponding score in the good-outcome group (Fig 6). The assumption is that the larger a score ratio deviates from one, the more likely a pathway is involved in a process that determines outcome.

The scores of the pathways in the bad-outcome group are consistently higher than the corresponding scores in the good-outcome group, with the exceptions of the MAPK signaling and cell cycle pathways yielding score ratios respectively equal to 0.97 and 0.94. The highest score ratios are found for *Jak-STAT signaling*, *ECM receptor interaction*, *cAMP signaling*, *TGF-Beta signaling and estrogen signaling*. S14–S18 Figs illustrate that single (epi-)genetic aberrations cannot be easily linked to the differential scoring of these 5 pathways, except for slight increases in MYC, CACNA1D (a Calcium channel) and KRAS amplification, and infrequent CREBBP (a transcription factor activator) mutations. Still, all five pathways are involved in tumor development and proliferation. Jak-STAT signaling is since long linked to cell migration and survival in ovarian cancer [36] and often, the expression of, or the responsiveness to TGF-Beta is lost, leading to cell proliferation [37]. Furthermore, it is known that the disruption of the extra cellular matrix (ECM interaction) can lead to the overproduction of growth factors that promote abnormal proliferation [38]. Estrogen signaling is thought to be involved in the establishment of a pro-tumorigenic micro-environment, and the long-term use of estrogen-only hormone replacements is linked to ovarian cancer development [39]. Finally, cAMP signaling is involved in cell survival in ovarian cancer and the presence of mutant CREBBP proteins can lead to tumorigenesis [40].

Interestingly, when all pathways are considered instead of only the pathways that are important for the bad-outcome patient group, the ratio score ranking (S13 Fig) is topped by the *Notch signaling* pathway event though this pathway is not high-scored for any patient group. As mentioned above, Notch signaling is involved in embryonic development, proliferation, differentiation and apoptosis, and is implicated in ovarian (and many other) cancers [16,41,42]. Its large score ratio could be attributed to the differential amplification of DLL3 (S11 Fig), which recently has been reported to be the second most altered gene in the NOTCH3 pathway [43].

## Conclusions

We have presented a new pathway importance ranking strategy that allows for the integration of any dataset that can be cast in a binary format. The method was evaluated on two datasets retrieved from TCGA, containing gene expression, mutation, copy number and methylation data for breast and ovarian tumors.

By applying the method to the well-studied breast cancer TCGA dataset, we demonstrated the method’s potential to identify key pathways, involved in breast cancer development, that are important for all molecular subtypes. The method recapitulates to a large extent what is known about breast cancer pathway activity. Interestingly, sometimes pathways were deemed equally important for different subtypes, yet the underlying (epi)-genetic disturbances were diverse. Furthermore, next to prioritizing universally high-scoring pathways, the pathway ranking method was able to identify subtype-specific pathways. Often the aggregate score of a pathway could not be motivated by a single mutation, copy number or methylation alteration, but rather by a combination of genetic and epi-genetic disturbances. This suggests that the integration of all data through a network of known gene interactions is an essential step in tumor analysis.

The analysis of ovarian tumors confirmed the method’s ability to correctly identify key pathways, irrespective of survival-based tumor subtypes. A differential analysis of survival-based subtypes revealed several pathways with higher importance for the bad-outcome patient group than for the good-outcome patient group. Many of the pathways exhibiting higher importance for the bad-outcome patient group could be related to tumor proliferation and survival.

## Supporting Information

S1 Fig Comparison of scores obtained by two permutation strategies. Scores for shuffled pathways (10000 permutations, X-axis) are plotted against the scores obtained after shuffling the gene labels of the input datasets (100 permutations, Y-axis). The correlation is > 0.99.
(TIF)

S2 Fig Cell cycle pathway summary for breast cancer. mRNA gene expression, mutation pattern, copy number status and methylation pattern for the genes of the cell cycle KEGG pathway (hsa4110). Red = high value/presence, blue = low value. Methylation data are rescaled to the interval [0,1]. Genes are sorted according to the significance of a Kruskal-Wallis test, with the subtype as categorical factor. Maximum 30 genes per data type are shown.
(TIF)

S3 Fig PI3K-Akt signaling pathway summary for breast cancer. mRNA gene expression, mutation pattern, copy number status and methylation pattern for the genes of the PI3K-Akt signaling KEGG pathway (hsa4151). Red = high value/presence, blue = low value. Methylation data are rescaled to the interval [0,1]. Genes are sorted according to the significance of a Kruskal-Wallis test, with the subtype as categorical factor. Maximum 30 genes per data type are shown.
(TIF)

S4 Fig Jak-STAT signaling pathway summary for breast cancer. mRNA gene expression, mutation pattern, copy number status and methylation pattern for the genes of the Jak-STAT signaling KEGG pathway (hsa4630). Red = high value/presence, blue = low value. Methylation data are rescaled to the interval [0,1]. Genes are sorted according to the significance of a Kruskal-Wallis test, with the subtype as categorical factor. Maximum 30 genes per data type are shown.
(TIF)

S5 Fig TGF-Beta signaling pathway summary for breast cancer. mRNA gene expression, mutation pattern, copy number status and methylation pattern for the genes of the TGF-Beta signaling KEGG pathway (hsa4350). Red = high value/presence, blue = low value. Methylation data are rescaled to the interval [0,1]. Genes are sorted according to the significance of a Kruskal-Wallis test, with the subtype as categorical factor. Maximum 30 genes per data type are shown.
(TIF)

S6 Fig p53 signaling pathway summary for breast cancer. mRNA gene expression, mutation pattern, copy number status and methylation pattern for the genes of the p53 signaling KEGG pathway (hsa4115). Red = high value/presence, blue = low value. Methylation data are rescaled to the interval [0,1]. Genes are sorted according to the significance of a Kruskal-Wallis test, with the subtype as categorical factor. Maximum 30 genes per data type are shown.
(TIF)

S7 Fig Toll-like receptor signaling pathway summary for breast cancer. mRNA gene expression, mutation pattern, copy number status and methylation pattern for the genes of the Toll-like receptor signaling KEGG pathway (hsa4620). Red = high value/presence, blue = low value. Methylation data are rescaled to the interval [0,1]. Genes are sorted according to the significance of a Kruskal-Wallis test, with the subtype as categorical factor. Maximum 30 genes per data type are shown.
(TIF)

S8 Fig ErbB signaling pathway summary for breast cancer. mRNA gene expression, mutation pattern, copy number status and methylation pattern for the genes of the ErbB signaling KEGG pathway (hsa4012). Red = high value/presence, blue = low value. Methylation data are rescaled to the interval [0,1]. Genes are sorted according to the significance of a Kruskal-Wallis test, with the subtype as categorical factor. Maximum 30 genes per data type are shown.
(TIF)

S9 Fig MAPK signaling pathway summary for breast cancer. mRNA gene expression, mutation pattern, copy number status and methylation pattern for the genes of the MAPK signaling KEGG pathway (hsa4010). Red = high value/presence, blue = low value. Methylation data are rescaled to the interval [0,1]. Genes are sorted according to the significance of a Kruskal-Wallis test, with the subtype as categorical factor. Maximum 30 genes per data type are shown.
(TIF)

S10 Fig AMPK signaling pathway summary for breast cancer. mRNA gene expression, mutation pattern, copy number status and methylation pattern for the genes of the AMPK signaling KEGG pathway (hsa4152). Red = high value/presence, blue = low value. Methylation data are rescaled to the interval [0,1]. Genes are sorted according to the significance of a Kruskal-Wallis test, with the subtype as categorical factor. Maximum 30 genes per data type are shown.
(TIF)

S11 Fig Notch signaling pathway summary for ovarian cancer. mRNA gene expression, mutation pattern, copy number status and methylation pattern for the genes of the Notch signaling KEGG pathway (hsa4330). Red = high value/presence, blue = low value. Methylation data are rescaled to the interval [0,1]. Genes are sorted according to the significance of a Kruskal-Wallis test, with the subtype as categorical factor (* = p<0.05, ** = p<0.01, *** = p<0.001). No FDR correction was applied. Maximum 30 genes per data type are shown.
(TIF)

S12 Fig WNT signaling pathway summary for ovarian cancer. mRNA gene expression, mutation pattern, copy number status and methylation pattern for the genes of the WNT signaling KEGG pathway (hsa4310). Red = high value/presence, blue = low value. Methylation data are rescaled to the interval [0,1]. Genes are sorted according to the significance of a Kruskal-Wallis test, with the subtype as categorical factor (* = p<0.05, ** = p<0.01, *** = p<0.001). No FDR correction was applied. Maximum 30 genes per data type are shown.
(TIF)

S13 Fig Ratio of bad-outcome pathway scores and the corresponding good-outcome scores for 100 pathways. A ratio of ‘1’ indicates that the pathway scores equally high for patients in the bad-outcome group and patients in the good-outcome group. Values larger than 1 indicate higher pathway importance / activity for the bad-outcome group.
(TIF)

S14 Fig Jak-STAT signaling pathway summary for ovarian cancer. mRNA gene expression, mutation pattern, copy number status and methylation pattern for the genes of the Jak-STAT signaling KEGG pathway (hsa4630). Red = high value/presence, blue = low value. Methylation data are rescaled to the interval [0,1]. Genes are sorted according to the significance of a Kruskal-Wallis test, with the subtype as categorical factor (* = p<0.05, ** = p<0.01, *** = p<0.001). No FDR correction was applied. Maximum 30 genes per data type are shown.
(TIF)

S15 Fig ECM-receptor interaction pathway summary for ovarian cancer. mRNA gene expression, mutation pattern, copy number status and methylation pattern for the genes of the ECM-receptor interaction KEGG pathway (hsa4512). Red = high value/presence, blue = low value. Methylation data are rescaled to the interval [0,1]. Genes are sorted according to the significance of a Kruskal-Wallis test, with the subtype as categorical factor (* = p<0.05, ** = p<0.01, *** = p<0.001). No FDR correction was applied. Maximum 30 genes per data type are shown.
(TIF)

S16 Fig cAMP signaling pathway summary for ovarian cancer. mRNA gene expression, mutation pattern, copy number status and methylation pattern for the genes of the cAMP signaling KEGG pathway (hsa4024). Red = high value/presence, blue = low value. Methylation data are rescaled to the interval [0,1]. Genes are sorted according to the significance of a Kruskal-Wallis test, with the subtype as categorical factor (* = p<0.05, ** = p<0.01, *** = p<0.001). No FDR correction was applied. Maximum 30 genes per data type are shown.
(TIF)

S17 Fig TGF-Beta signaling pathway summary for ovarian cancer. mRNA gene expression, mutation pattern, copy number status and methylation pattern for the genes of the TGF-Beta signaling KEGG pathway (hsa4350). Red = high value/presence, blue = low value. Methylation data are rescaled to the interval [0,1]. Genes are sorted according to the significance of a Kruskal-Wallis test, with the subtype as categorical factor (* = p<0.05, ** = p<0.01, *** = p<0.001). No FDR correction was applied. Maximum 30 genes per data type are shown.
(TIF)

S18 Fig Estrogen signaling pathway summary for ovarian cancer. mRNA gene expression, mutation pattern, copy number status and methylation pattern for the genes of the Estrogen signaling KEGG pathway (hsa4915). Red = high value/presence, blue = low value. Methylation data are rescaled to the interval [0,1]. Genes are sorted according to the significance of a Kruskal-Wallis test, with the subtype as categorical factor (* = p<0.05, ** = p<0.01, *** = p<0.001). No FDR correction was applied. Maximum 30 genes per data type are shown.
(TIF)

S1 Table Mapping of the top-scoring pathways from the original PARADIGM study on KEGG pathways. (XLSX)

S2 Table Comparison of the proposed, network-based method (SIM) to a naïve frequency-based approach (FRQ). Pathway IDs correspond to KEGG identifiers.
(PDF)
