# Supplementary figures and images for: Pathway Relevance Ranking for Tumor Samples through Network-Based Data Integration
Source: PLoS One. 2015 Jul 28;10(7):e0133503. doi: 10.1371/journal.pone.0133503 (PMC4517887; doi:10.1371/journal.pone.0133503)

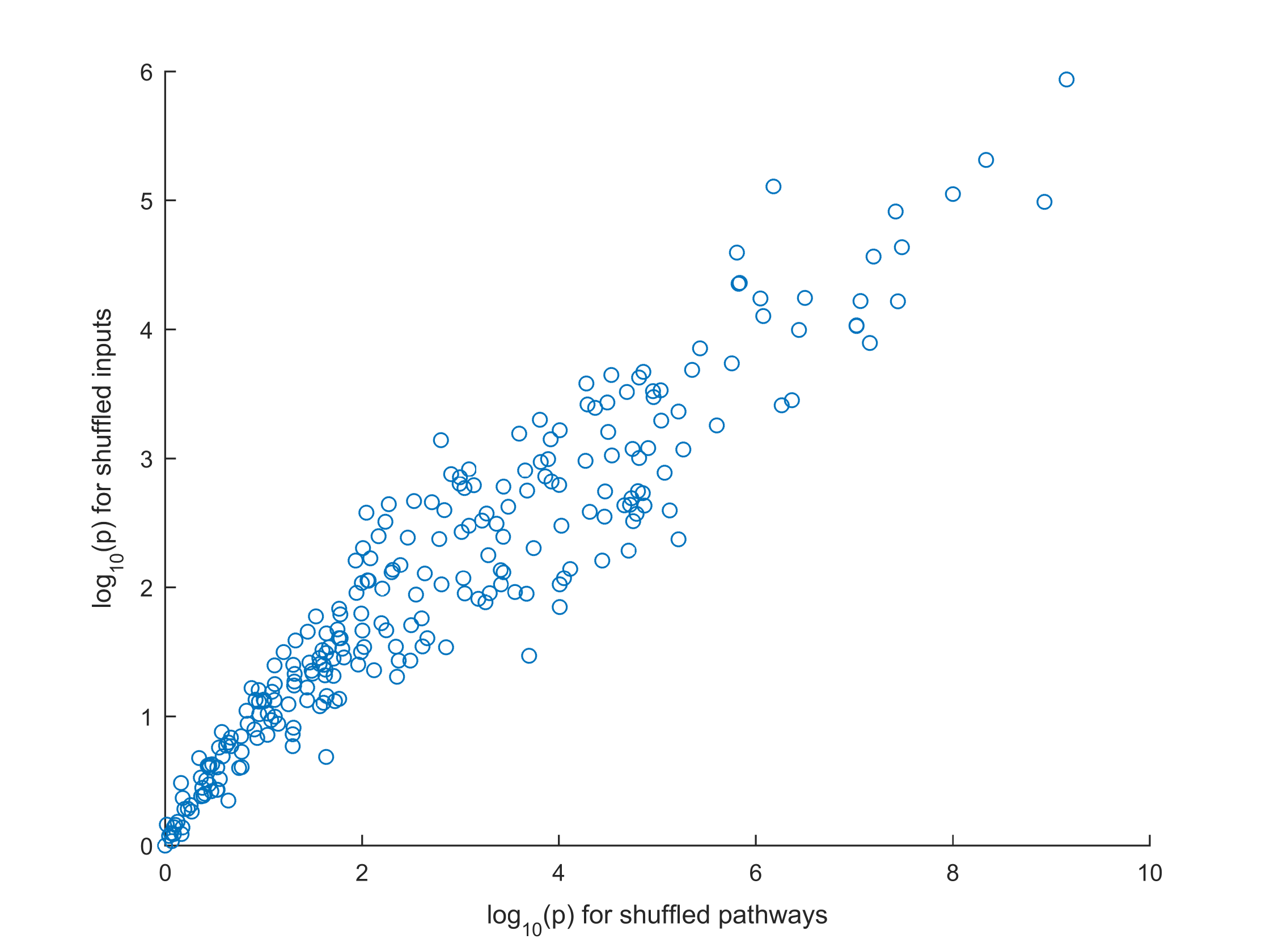

Supplement: S1 Fig — Scores for shuffled pathways (10000 permutations, X-axis) are plotted against the scores obtained after shuffling the gene labels of the input datasets (100 permutations, Y-axis). The correlation is > 0.99. (TIF) [file pone.0133503.s001.tif]

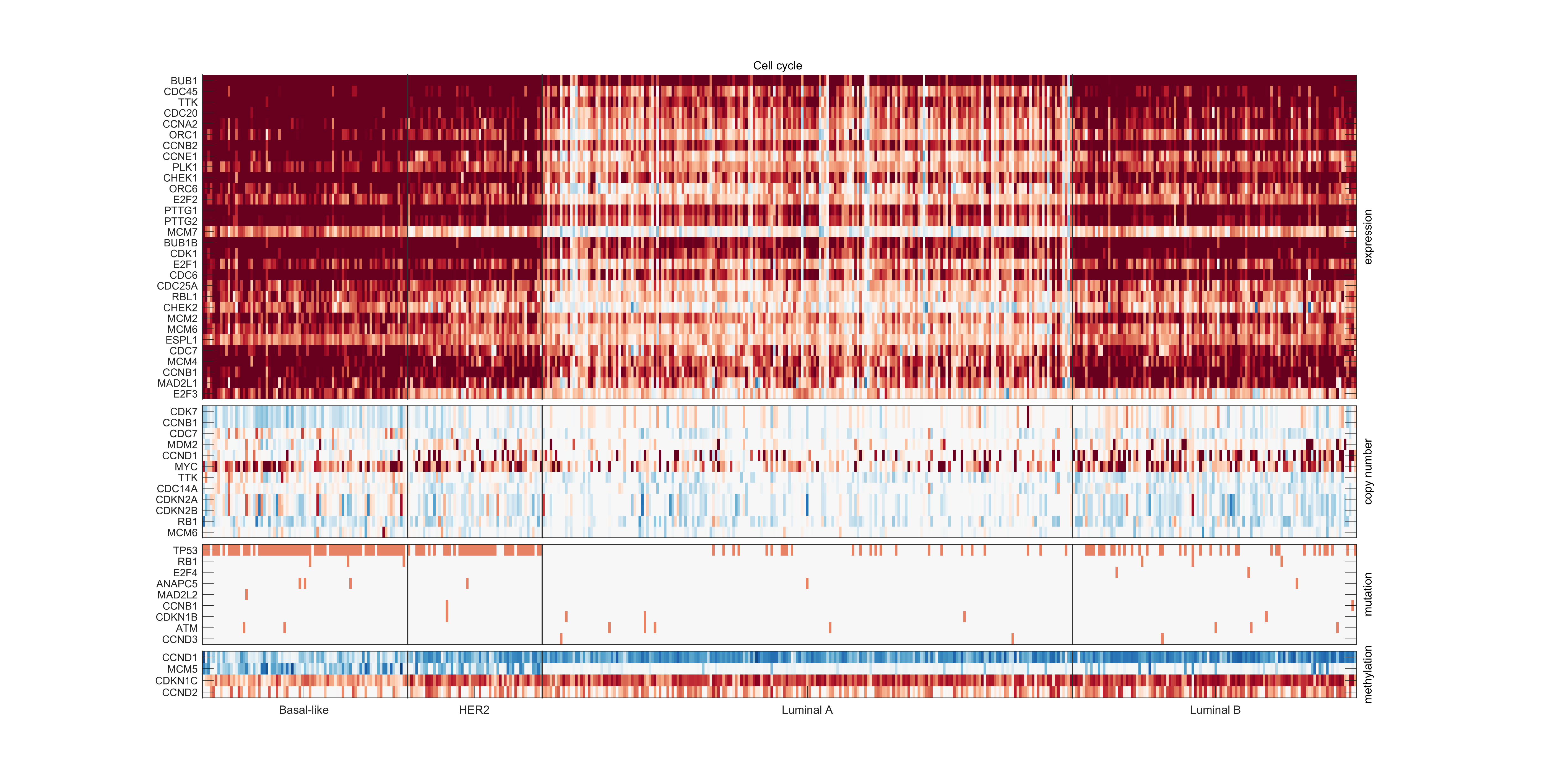

Supplement: S2 Fig — mRNA gene expression, mutation pattern, copy number status and methylation pattern for the genes of the cell cycle KEGG pathway (hsa4110). Red = high value/presence, blue = low value. Methylation data are rescaled to the interval [0,1]. Genes are sorted according to the significance of a Kruskal-Wallis test, with the subtype as categorical factor. Maximum 30 genes per data type are shown. (TIF) [file pone.0133503.s002.tif]

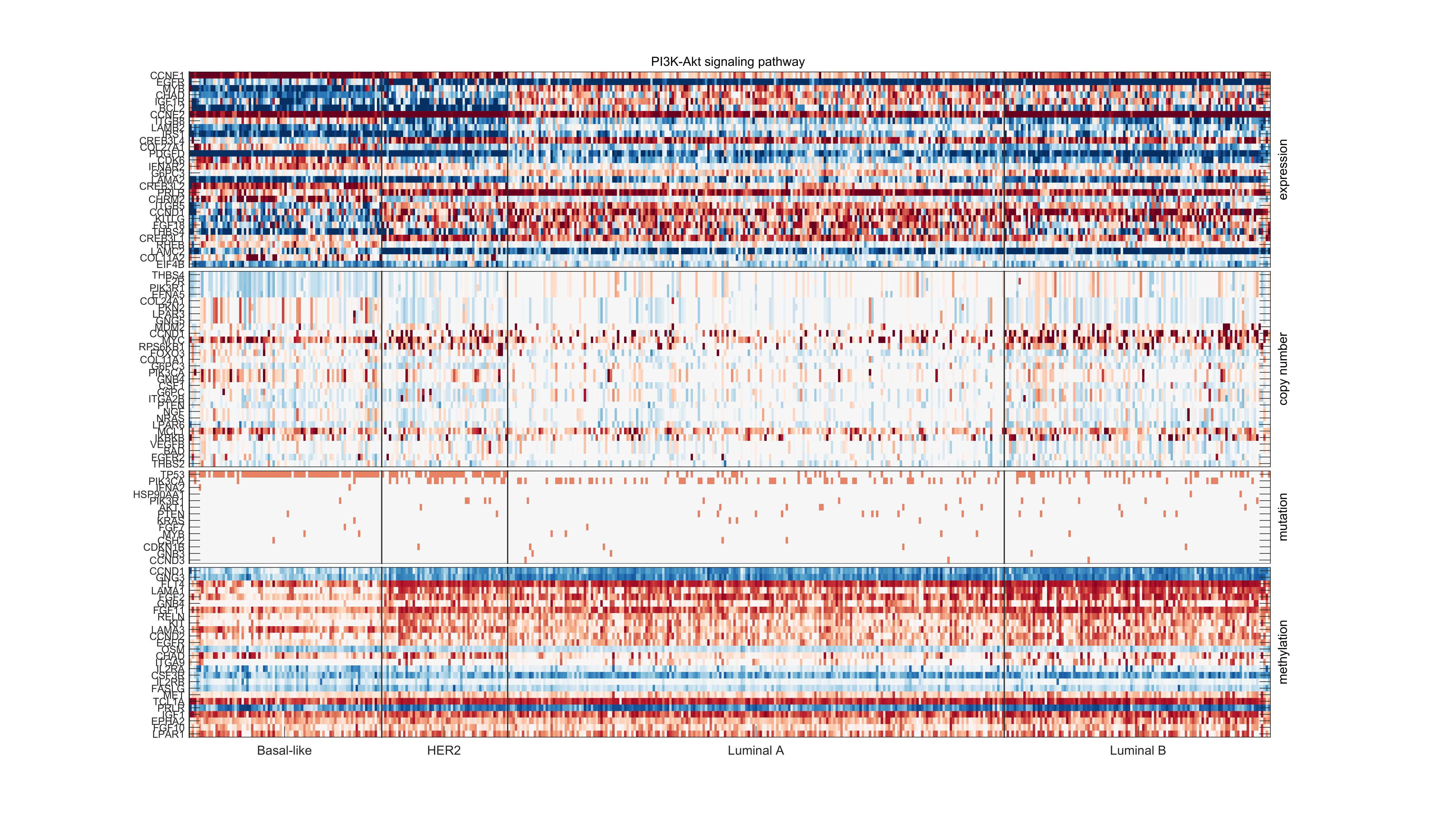

Supplement: S3 Fig — mRNA gene expression, mutation pattern, copy number status and methylation pattern for the genes of the PI3K-Akt signaling KEGG pathway (hsa4151). Red = high value/presence, blue = low value. Methylation data are rescaled to the interval [0,1]. Genes are sorted according to the significance of a Kruskal-Wallis test, with the subtype as categorical factor. Maximum 30 genes per data type are shown. (TIF) [file pone.0133503.s003.tif]

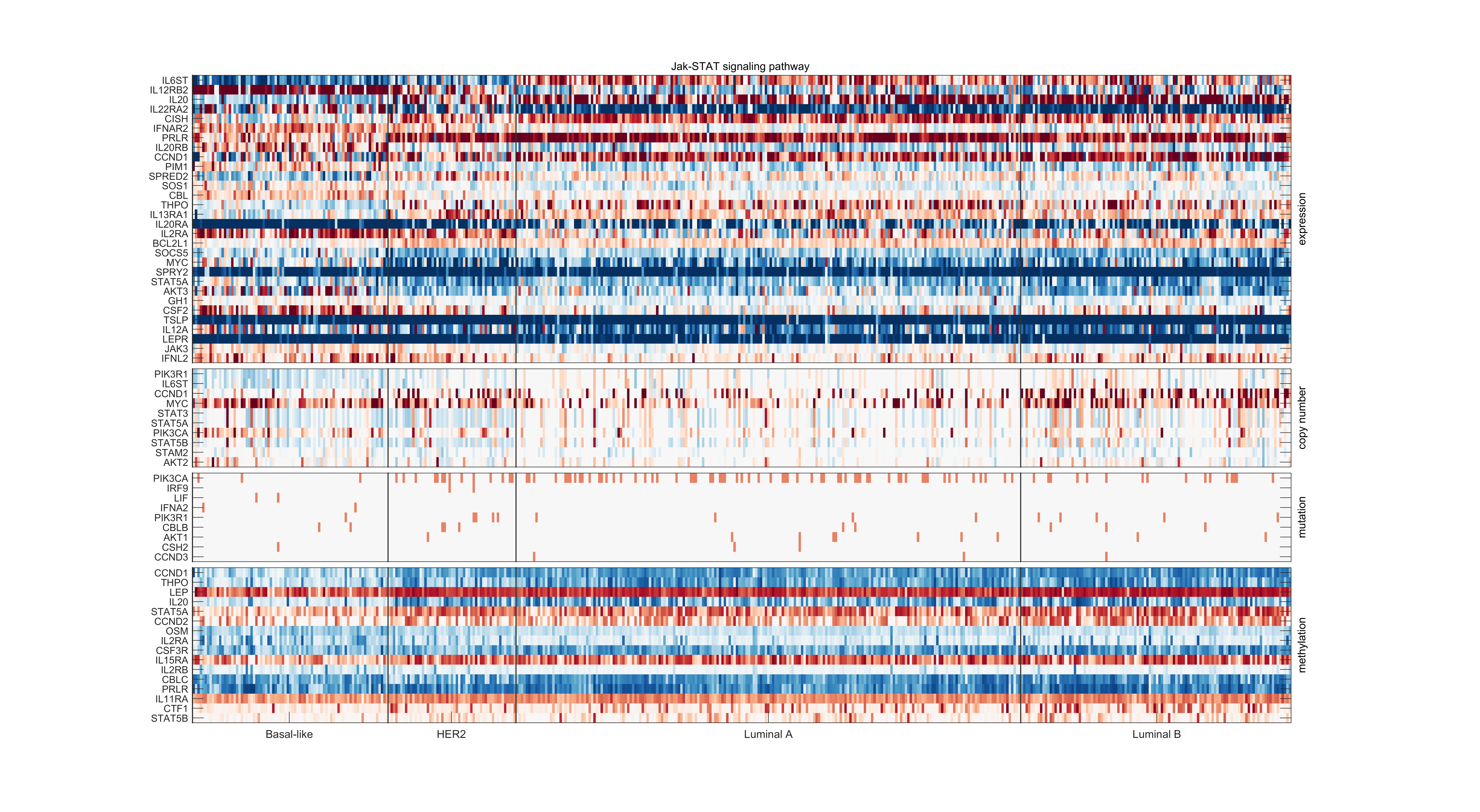

Supplement: S4 Fig — mRNA gene expression, mutation pattern, copy number status and methylation pattern for the genes of the Jak-STAT signaling KEGG pathway (hsa4630). Red = high value/presence, blue = low value. Methylation data are rescaled to the interval [0,1]. Genes are sorted according to the significance of a Kruskal-Wallis test, with the subtype as categorical factor. Maximum 30 genes per data type are shown. (TIF) [file pone.0133503.s004.tif]

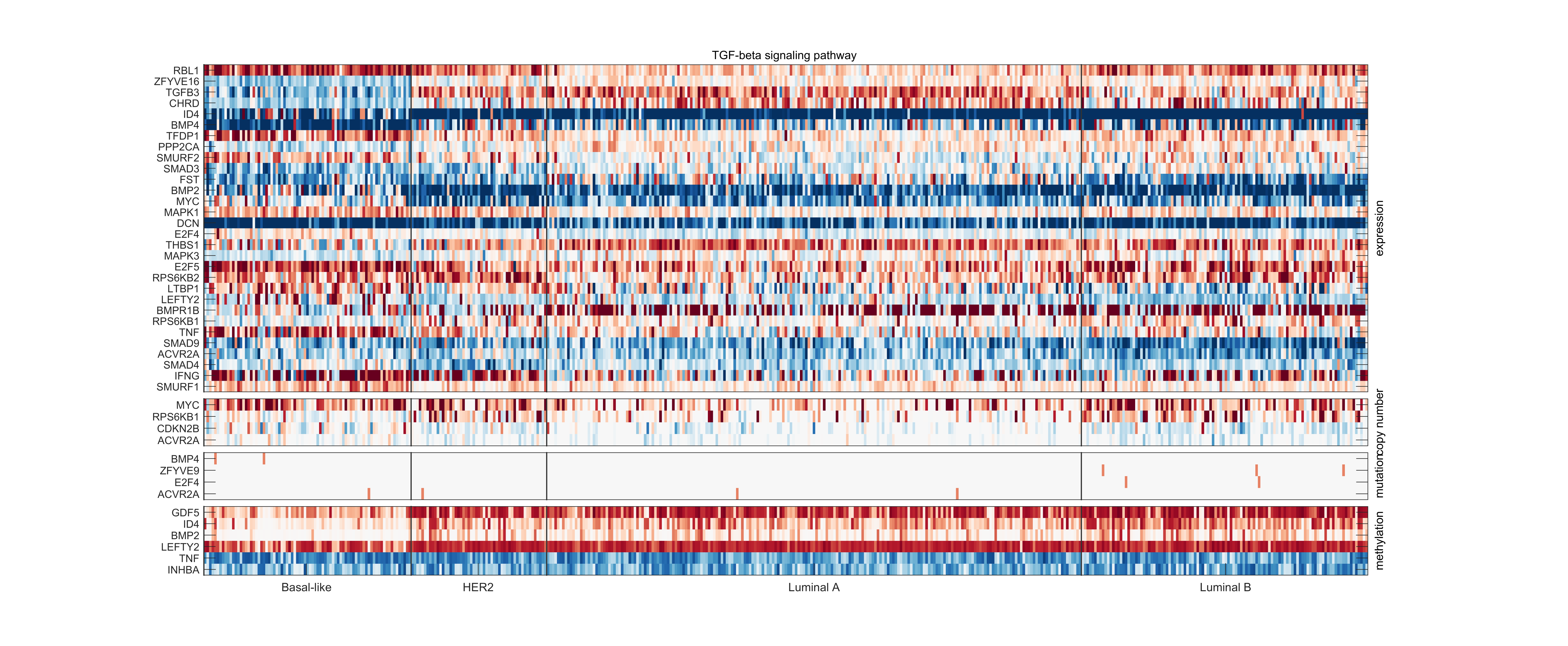

Supplement: S5 Fig — mRNA gene expression, mutation pattern, copy number status and methylation pattern for the genes of the TGF-Beta signaling KEGG pathway (hsa4350). Red = high value/presence, blue = low value. Methylation data are rescaled to the interval [0,1]. Genes are sorted according to the significance of a Kruskal-Wallis test, with the subtype as categorical factor. Maximum 30 genes per data type are shown. (TIF) [file pone.0133503.s005.tif]

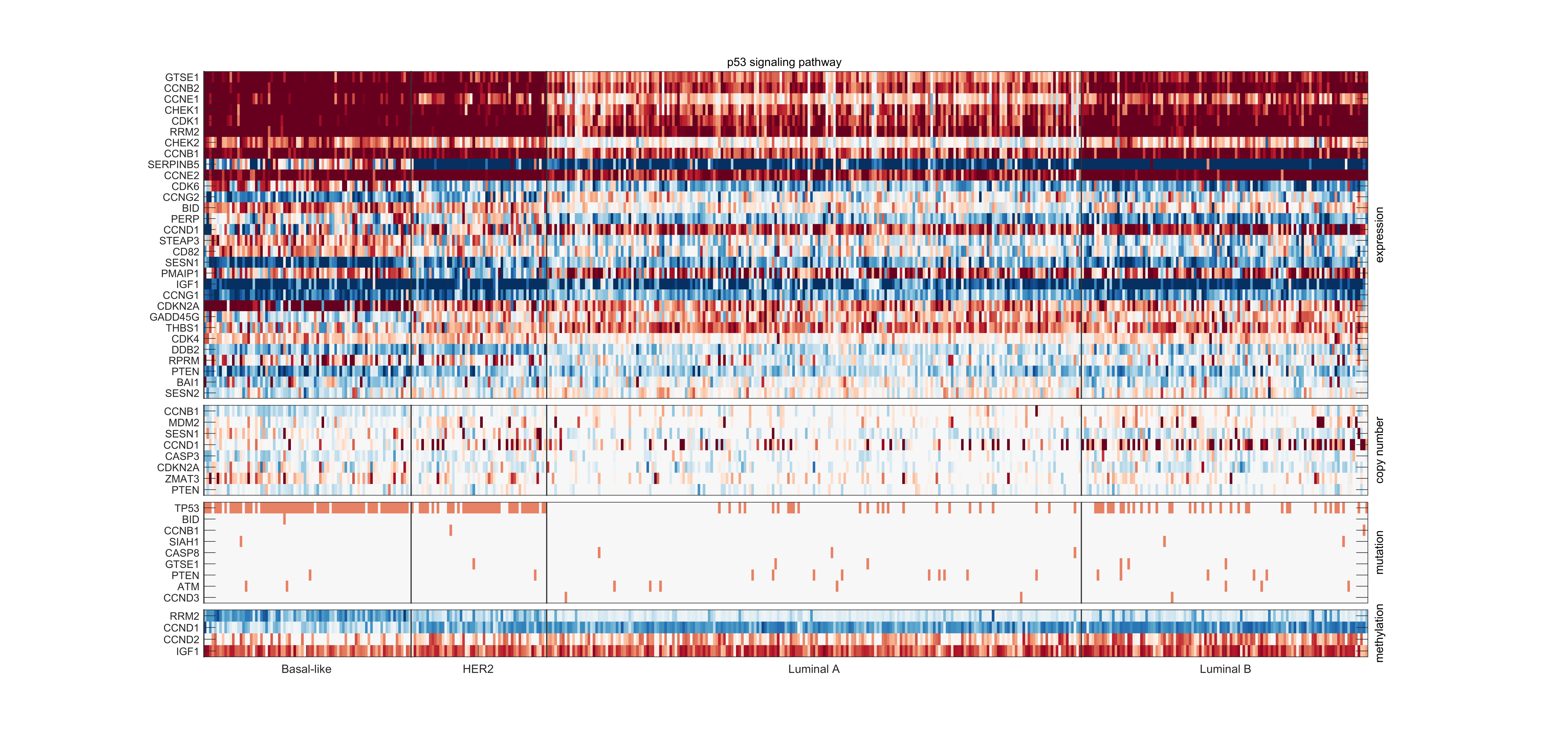

Supplement: S6 Fig — mRNA gene expression, mutation pattern, copy number status and methylation pattern for the genes of the p53 signaling KEGG pathway (hsa4115). Red = high value/presence, blue = low value. Methylation data are rescaled to the interval [0,1]. Genes are sorted according to the significance of a Kruskal-Wallis test, with the subtype as categorical factor. Maximum 30 genes per data type are shown. (TIF) [file pone.0133503.s006.tif]

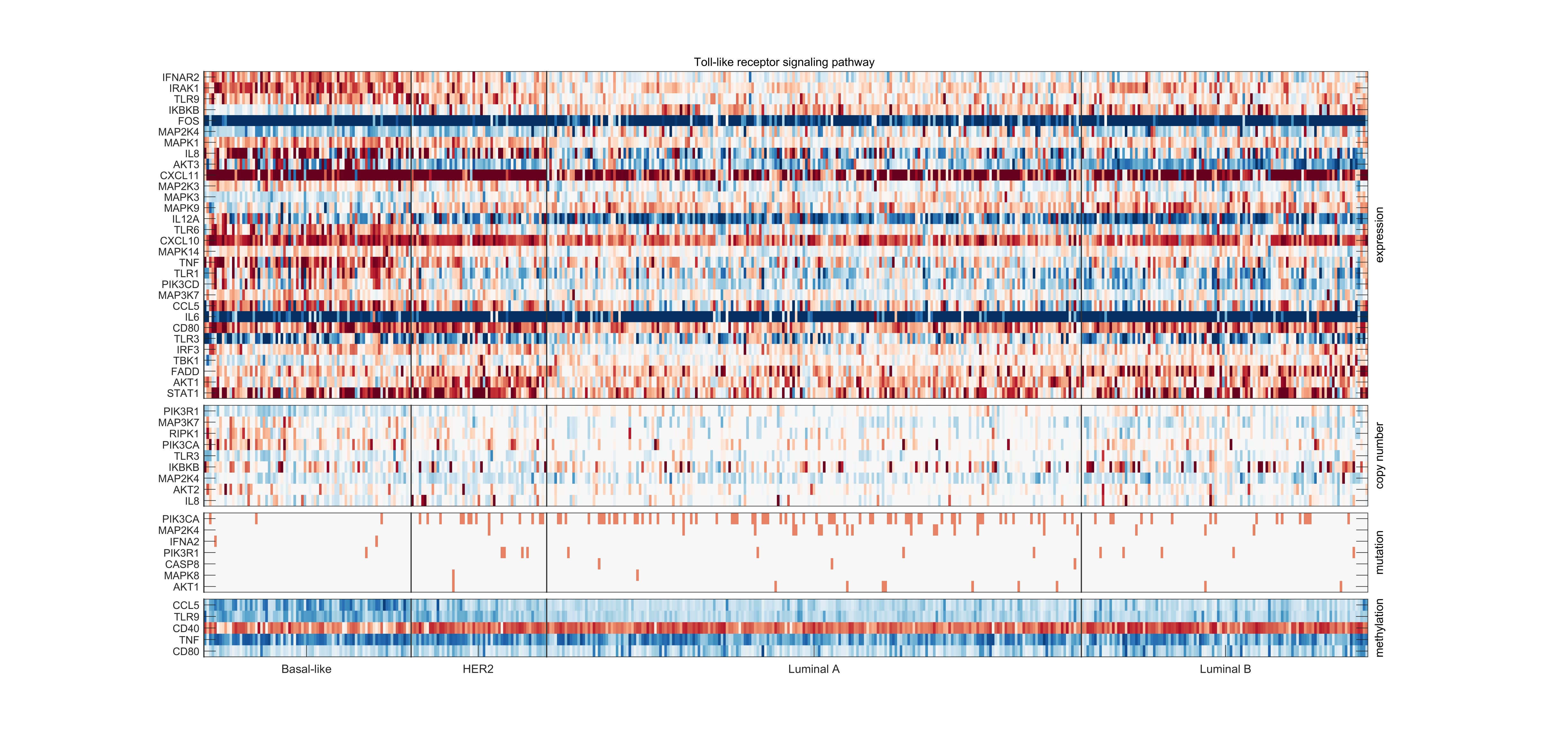

Supplement: S7 Fig — mRNA gene expression, mutation pattern, copy number status and methylation pattern for the genes of the Toll-like receptor signaling KEGG pathway (hsa4620). Red = high value/presence, blue = low value. Methylation data are rescaled to the interval [0,1]. Genes are sorted according to the significance of a Kruskal-Wallis test, with the subtype as categorical factor. Maximum 30 genes per data type are shown. (TIF) [file pone.0133503.s007.tif]

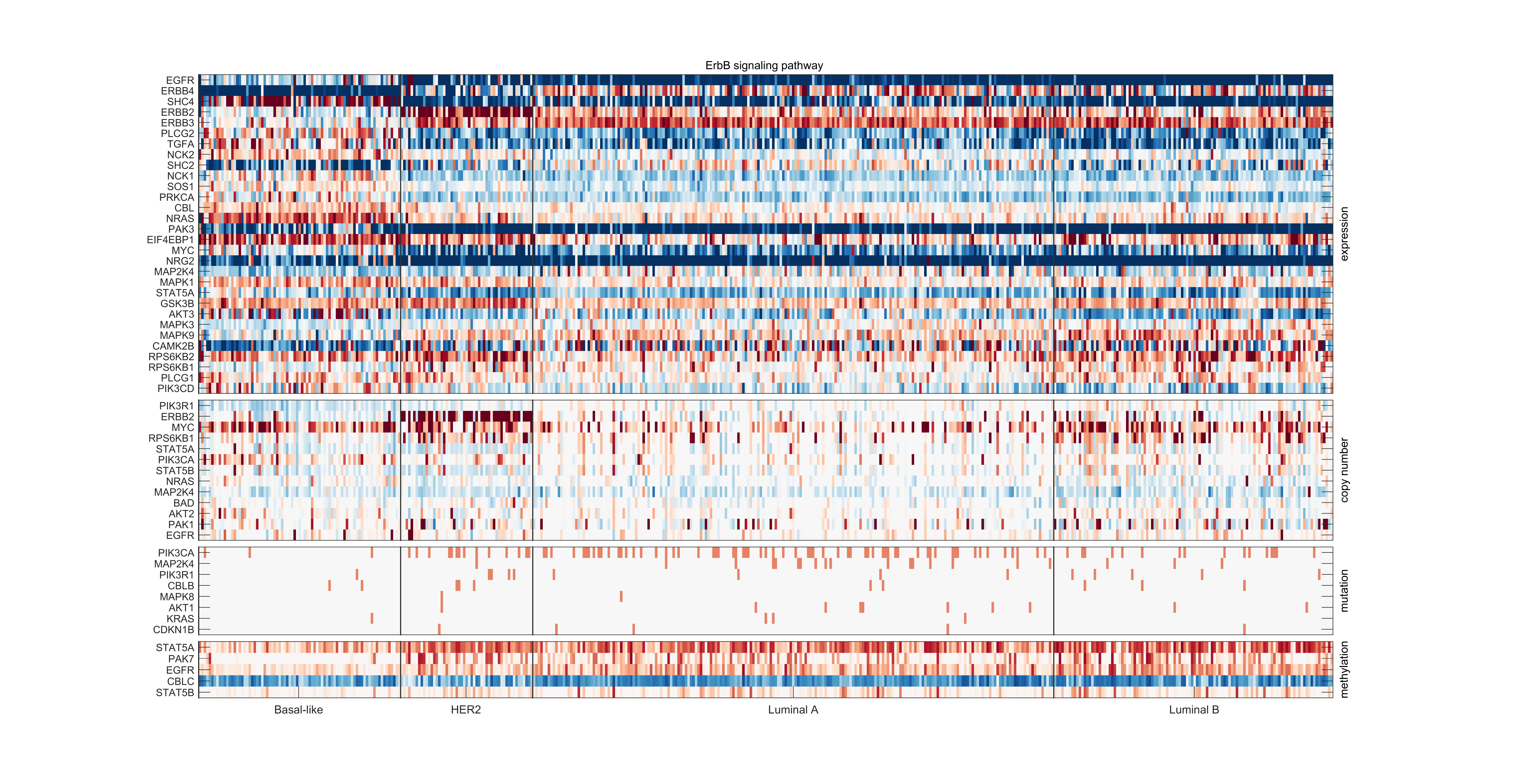

Supplement: S8 Fig — mRNA gene expression, mutation pattern, copy number status and methylation pattern for the genes of the ErbB signaling KEGG pathway (hsa4012). Red = high value/presence, blue = low value. Methylation data are rescaled to the interval [0,1]. Genes are sorted according to the significance of a Kruskal-Wallis test, with the subtype as categorical factor. Maximum 30 genes per data type are shown. (TIF) [file pone.0133503.s008.tif]

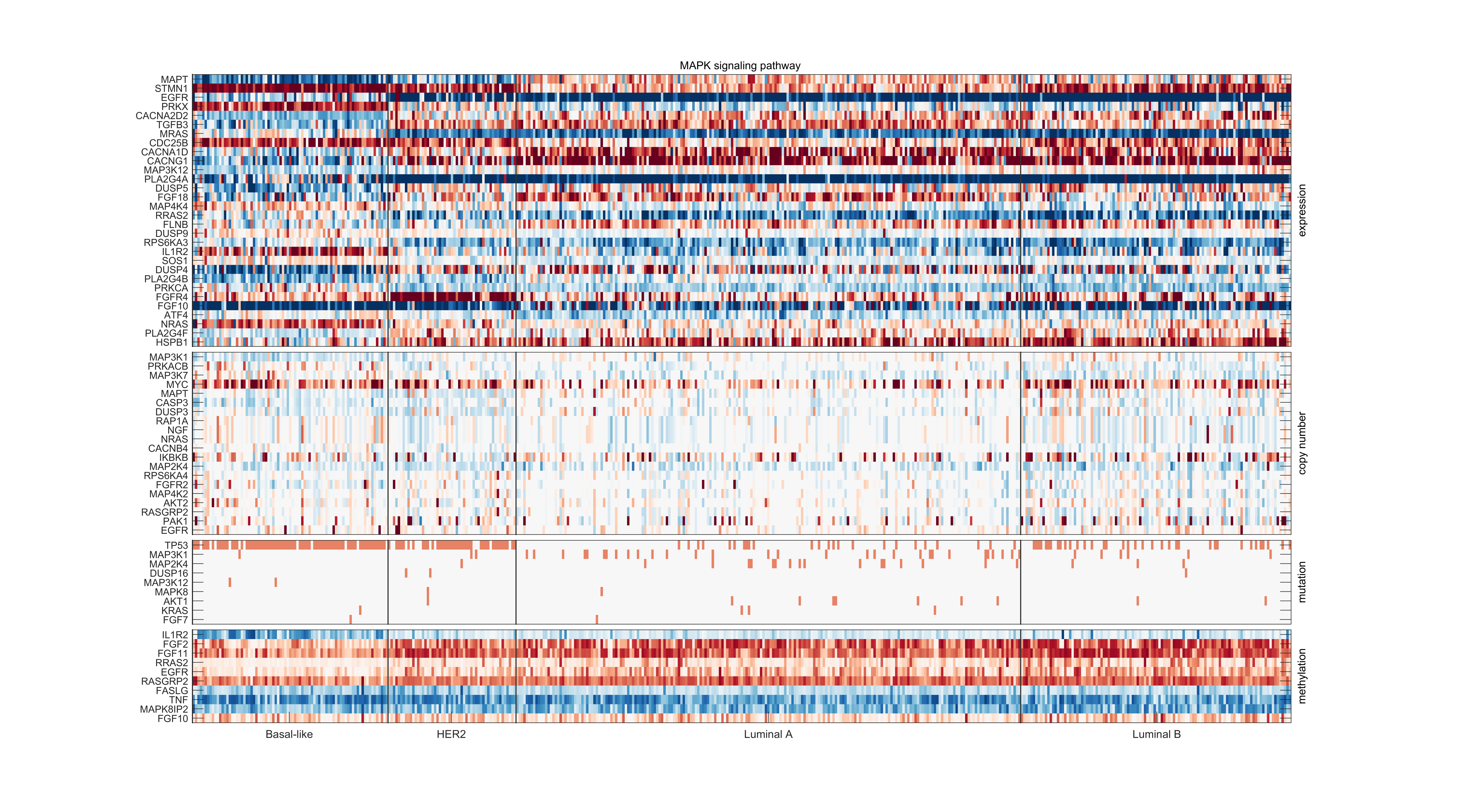

Supplement: S9 Fig — mRNA gene expression, mutation pattern, copy number status and methylation pattern for the genes of the MAPK signaling KEGG pathway (hsa4010). Red = high value/presence, blue = low value. Methylation data are rescaled to the interval [0,1]. Genes are sorted according to the significance of a Kruskal-Wallis test, with the subtype as categorical factor. Maximum 30 genes per data type are shown. (TIF) [file pone.0133503.s009.tif]

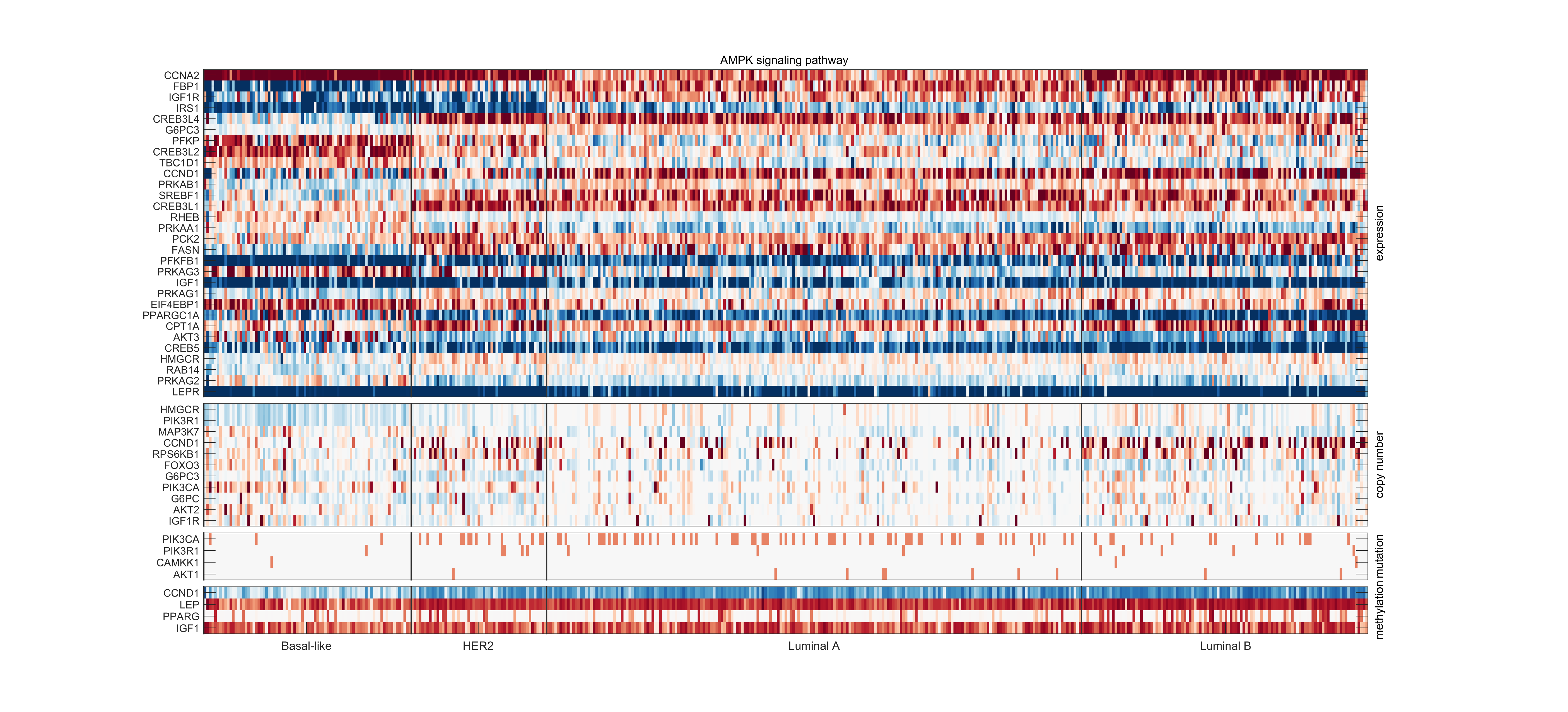

Supplement: S10 Fig — mRNA gene expression, mutation pattern, copy number status and methylation pattern for the genes of the AMPK signaling KEGG pathway (hsa4152). Red = high value/presence, blue = low value. Methylation data are rescaled to the interval [0,1]. Genes are sorted according to the significance of a Kruskal-Wallis test, with the subtype as categorical factor. Maximum 30 genes per data type are shown. (TIF) [file pone.0133503.s010.tif]

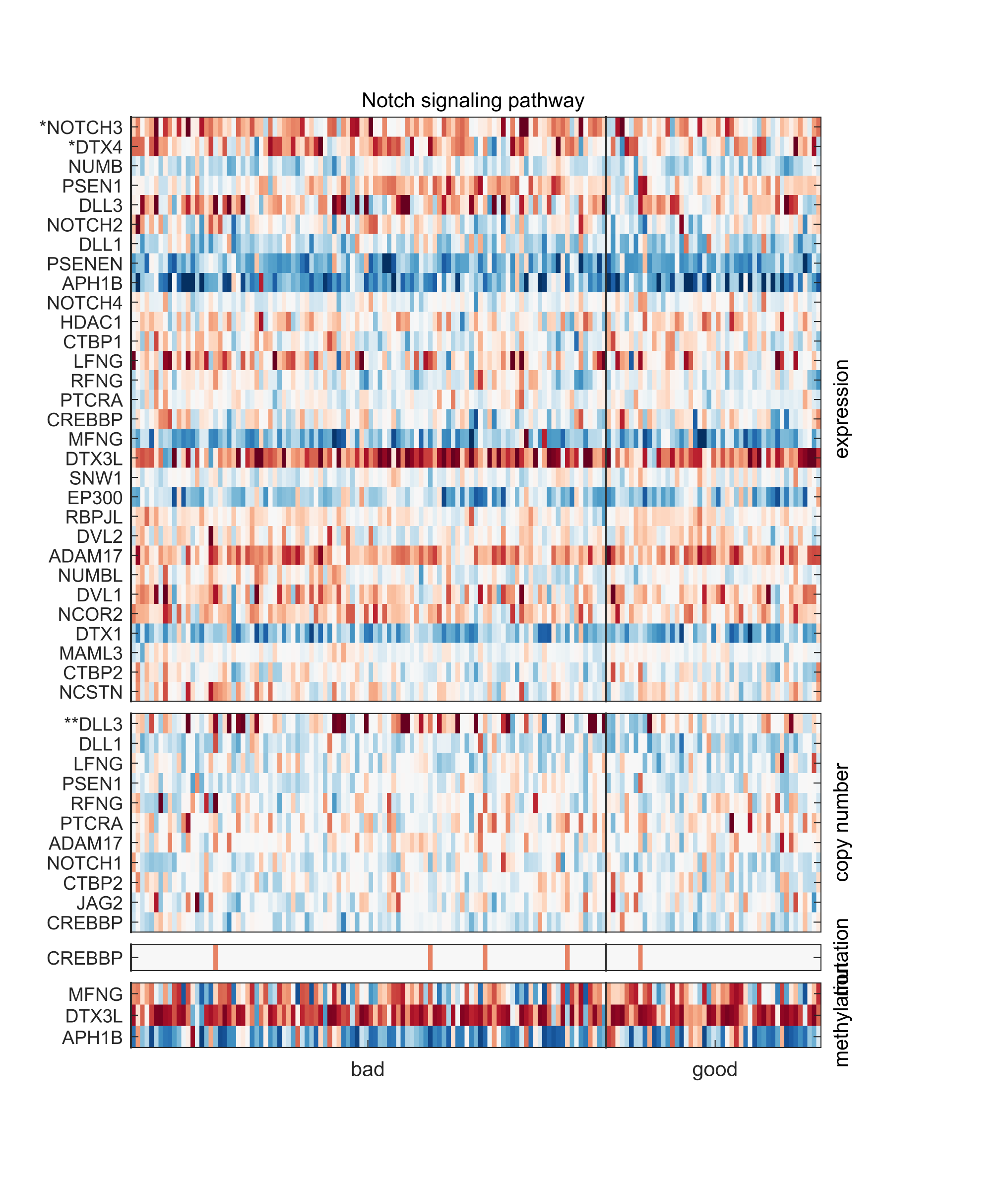

Supplement: S11 Fig — mRNA gene expression, mutation pattern, copy number status and methylation pattern for the genes of the Notch signaling KEGG pathway (hsa4330). Red = high value/presence, blue = low value. Methylation data are rescaled to the interval [0,1]. Genes are sorted according to the significance of a Kruskal-Wallis test, with the subtype as categorical factor (* = p<0.05, ** = p<0.01, *** = p<0.001). No FDR correction was applied. Maximum 30 genes per data type are shown. (TIF) [file pone.0133503.s011.tif]

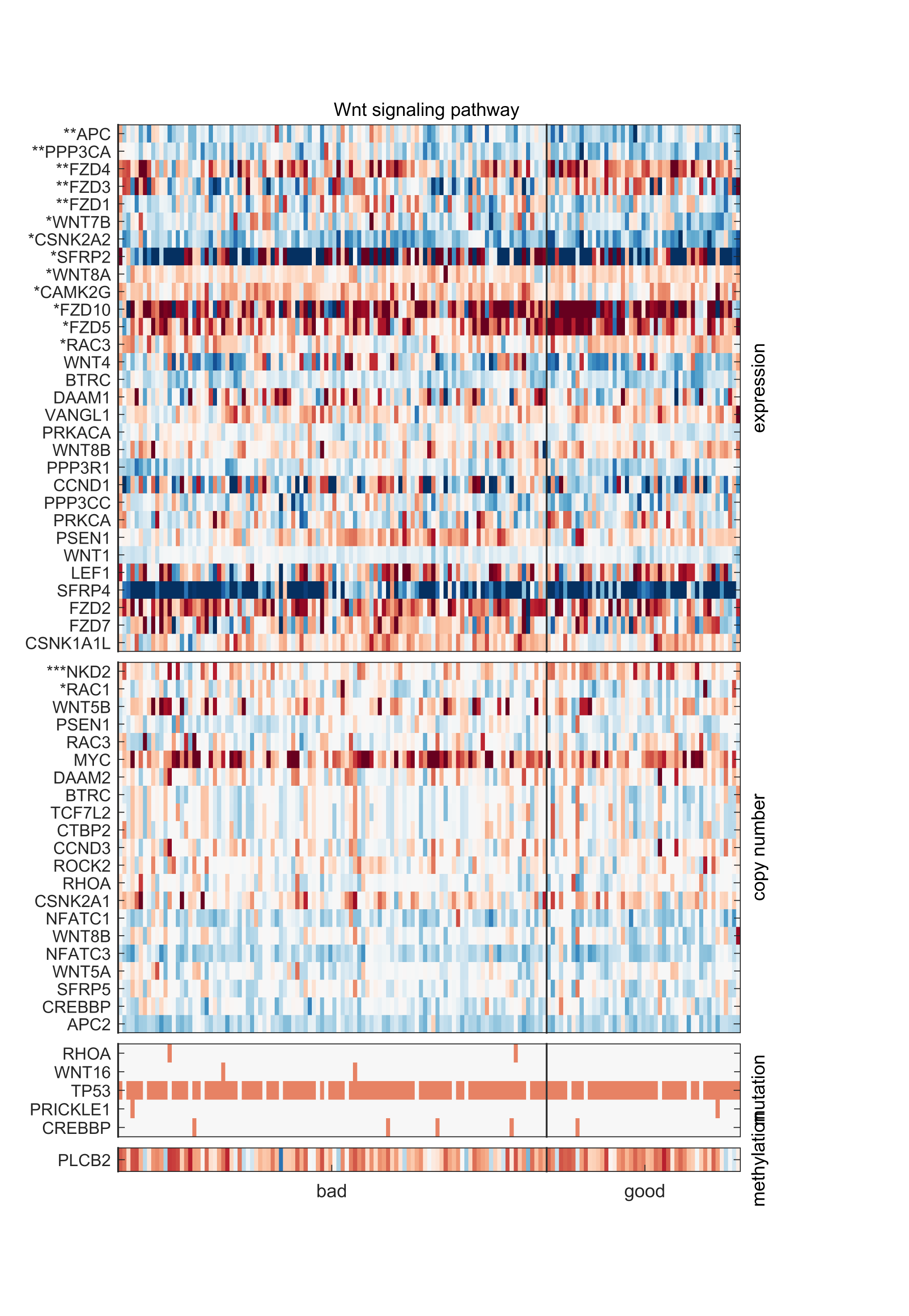

Supplement: S12 Fig — mRNA gene expression, mutation pattern, copy number status and methylation pattern for the genes of the WNT signaling KEGG pathway (hsa4310). Red = high value/presence, blue = low value. Methylation data are rescaled to the interval [0,1]. Genes are sorted according to the significance of a Kruskal-Wallis test, with the subtype as categorical factor (* = p<0.05, ** = p<0.01, *** = p<0.001). No FDR correction was applied. Maximum 30 genes per data type are shown. (TIF) [file pone.0133503.s012.tif]

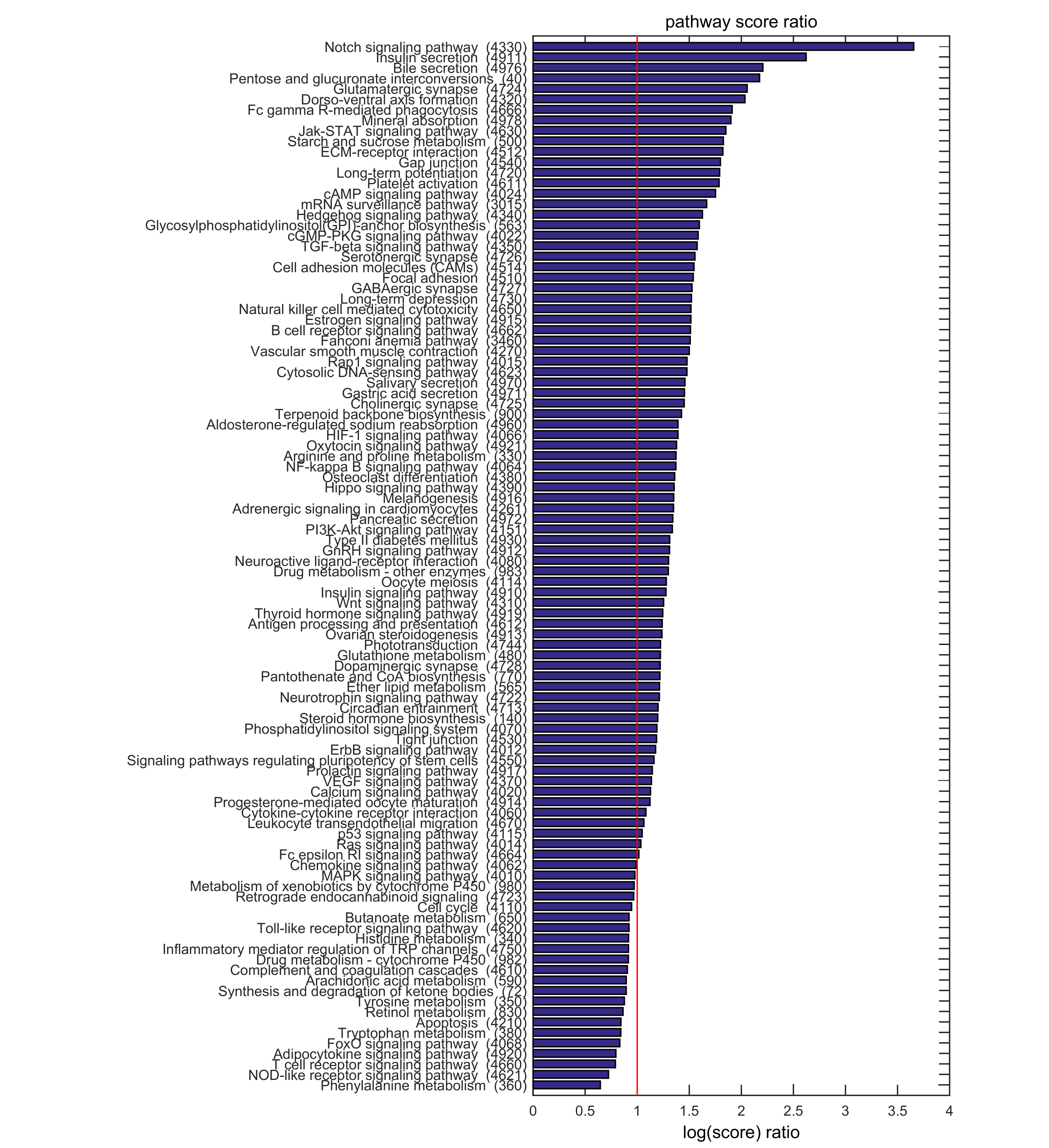

Supplement: S13 Fig — A ratio of ‘1’ indicates that the pathway scores equally high for patients in the bad-outcome group and patients in the good-outcome group. Values larger than 1 indicate higher pathway importance / activity for the bad-outcome group. (TIF) [file pone.0133503.s013.tif]

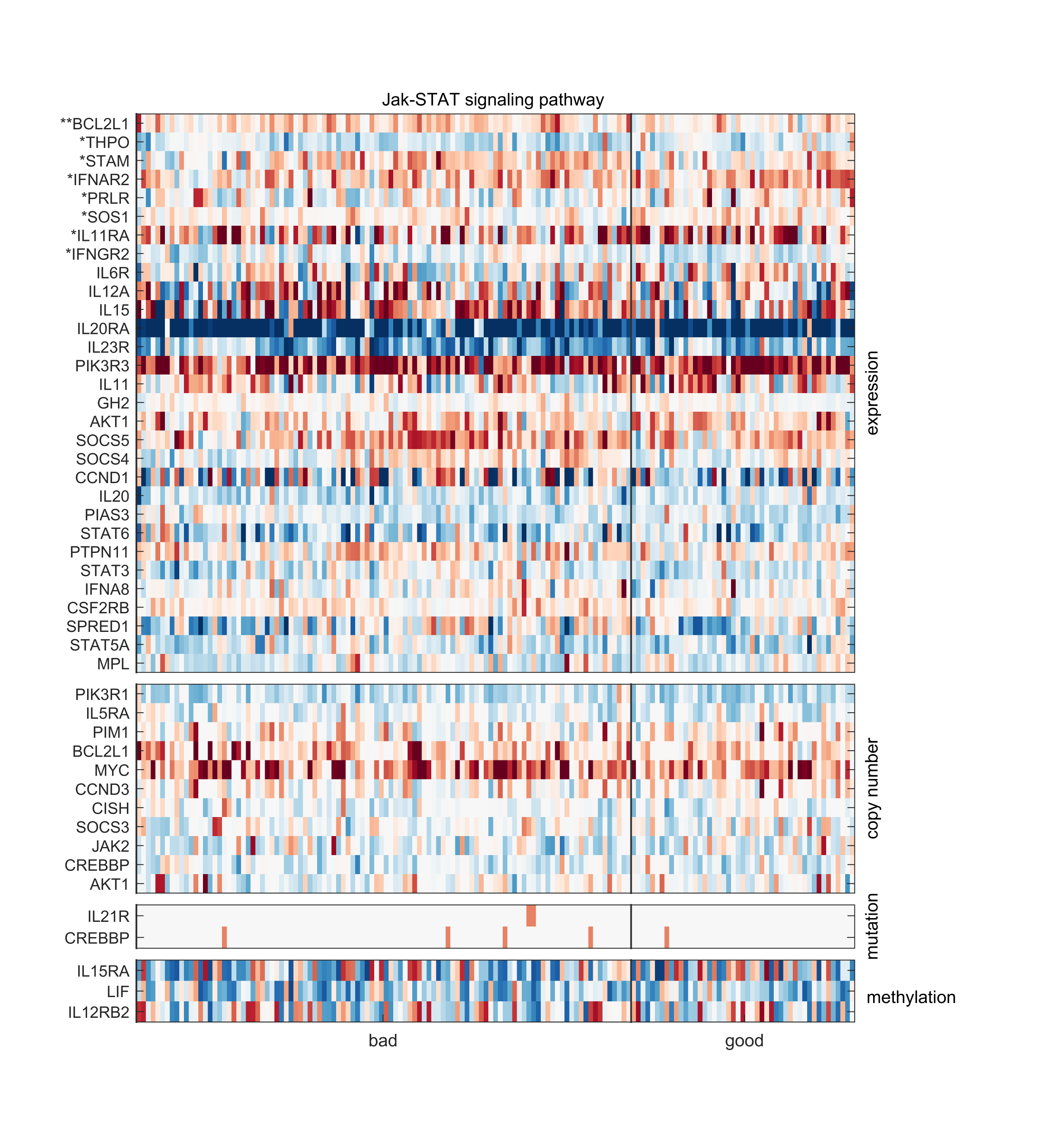

Supplement: S14 Fig — mRNA gene expression, mutation pattern, copy number status and methylation pattern for the genes of the Jak-STAT signaling KEGG pathway (hsa4630). Red = high value/presence, blue = low value. Methylation data are rescaled to the interval [0,1]. Genes are sorted according to the significance of a Kruskal-Wallis test, with the subtype as categorical factor (* = p<0.05, ** = p<0.01, *** = p<0.001). No FDR correction was applied. Maximum 30 genes per data type are shown. (TIF) [file pone.0133503.s014.tif]

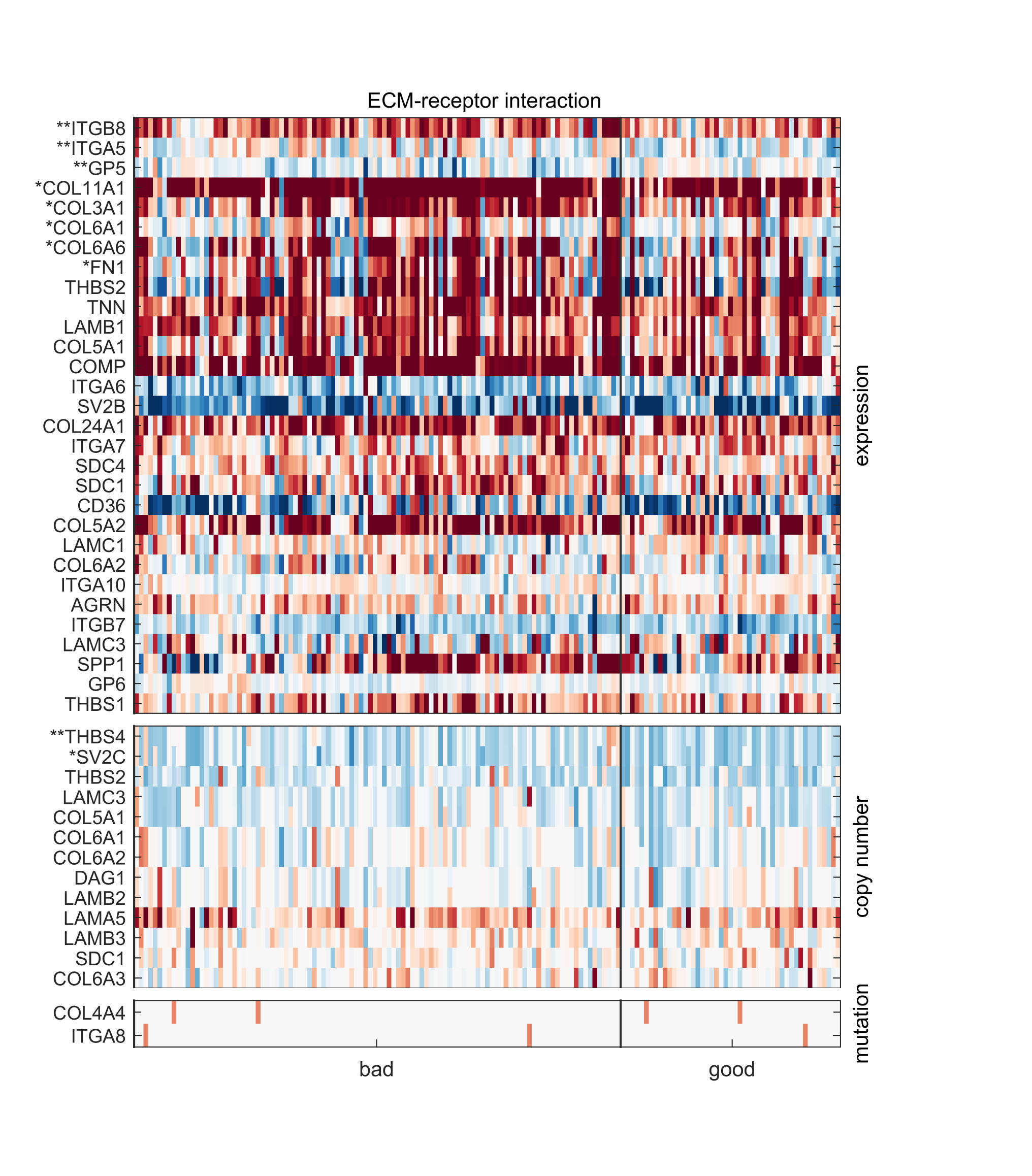

Supplement: S15 Fig — mRNA gene expression, mutation pattern, copy number status and methylation pattern for the genes of the ECM-receptor interaction KEGG pathway (hsa4512). Red = high value/presence, blue = low value. Methylation data are rescaled to the interval [0,1]. Genes are sorted according to the significance of a Kruskal-Wallis test, with the subtype as categorical factor (* = p<0.05, ** = p<0.01, *** = p<0.001). No FDR correction was applied. Maximum 30 genes per data type are shown. (TIF) [file pone.0133503.s015.tif]

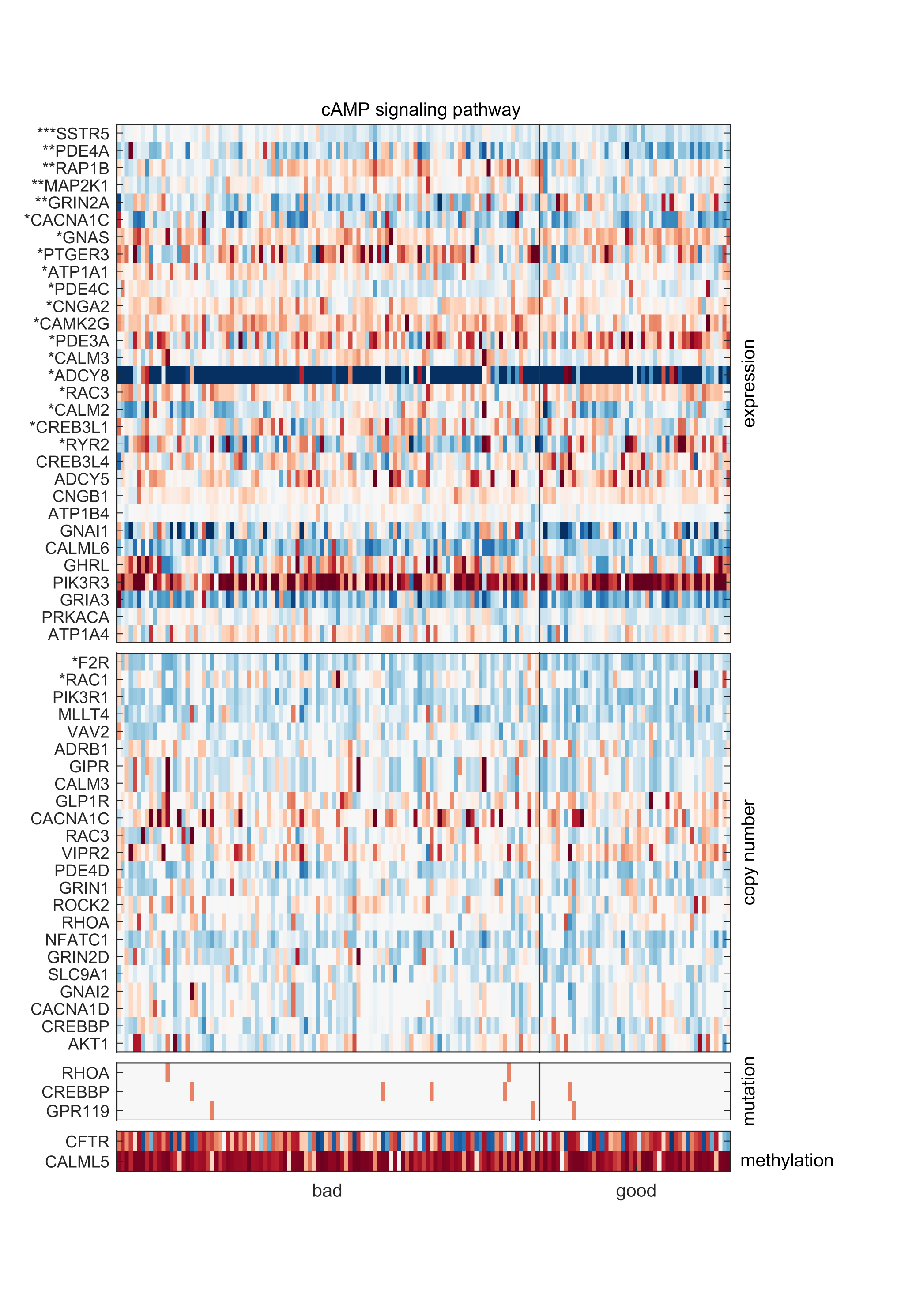

Supplement: S16 Fig — mRNA gene expression, mutation pattern, copy number status and methylation pattern for the genes of the cAMP signaling KEGG pathway (hsa4024). Red = high value/presence, blue = low value. Methylation data are rescaled to the interval [0,1]. Genes are sorted according to the significance of a Kruskal-Wallis test, with the subtype as categorical factor (* = p<0.05, ** = p<0.01, *** = p<0.001). No FDR correction was applied. Maximum 30 genes per data type are shown. (TIF) [file pone.0133503.s016.tif]

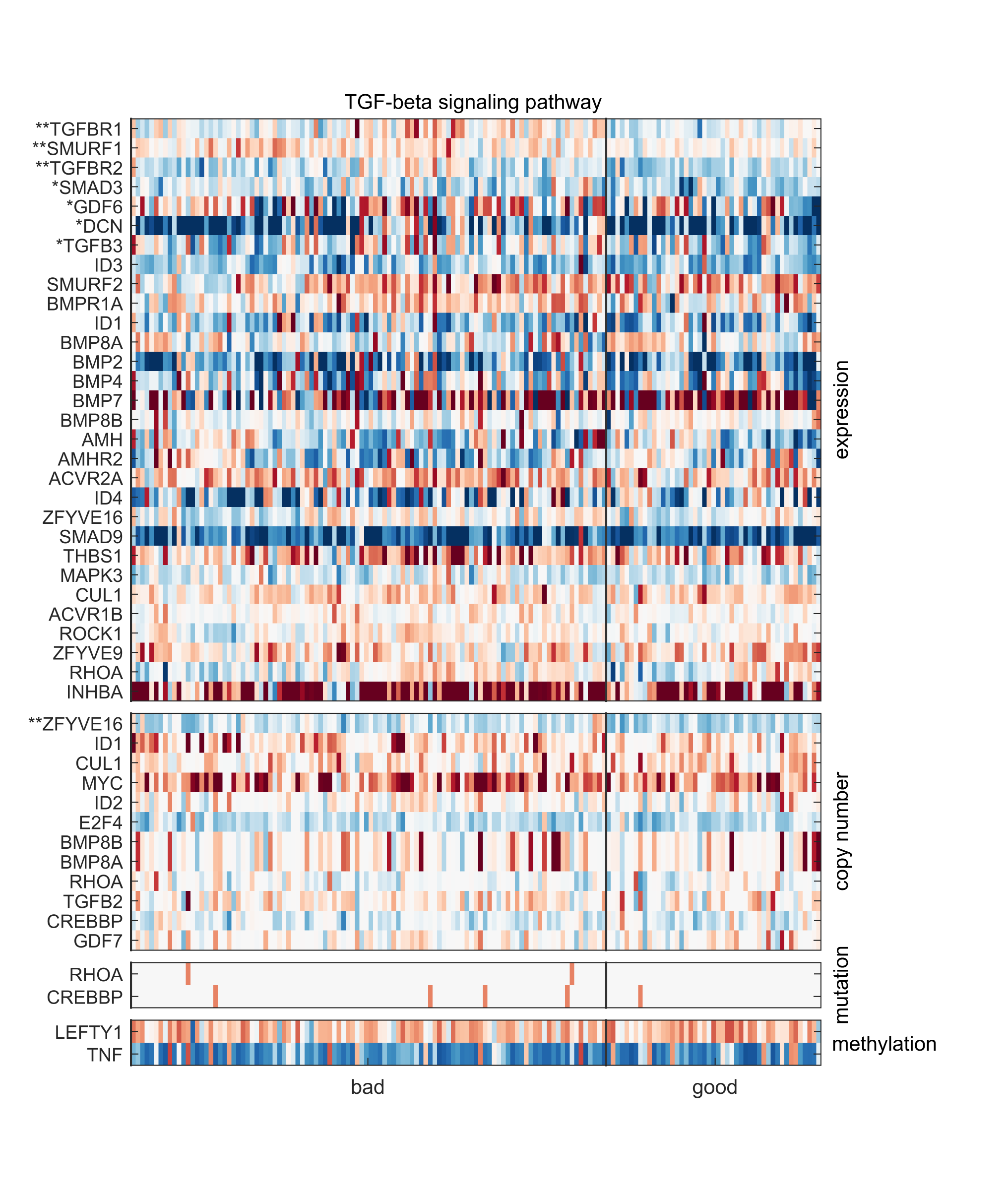

Supplement: S17 Fig — mRNA gene expression, mutation pattern, copy number status and methylation pattern for the genes of the TGF-Beta signaling KEGG pathway (hsa4350). Red = high value/presence, blue = low value. Methylation data are rescaled to the interval [0,1]. Genes are sorted according to the significance of a Kruskal-Wallis test, with the subtype as categorical factor (* = p<0.05, ** = p<0.01, *** = p<0.001). No FDR correction was applied. Maximum 30 genes per data type are shown. (TIF) [file pone.0133503.s017.tif]

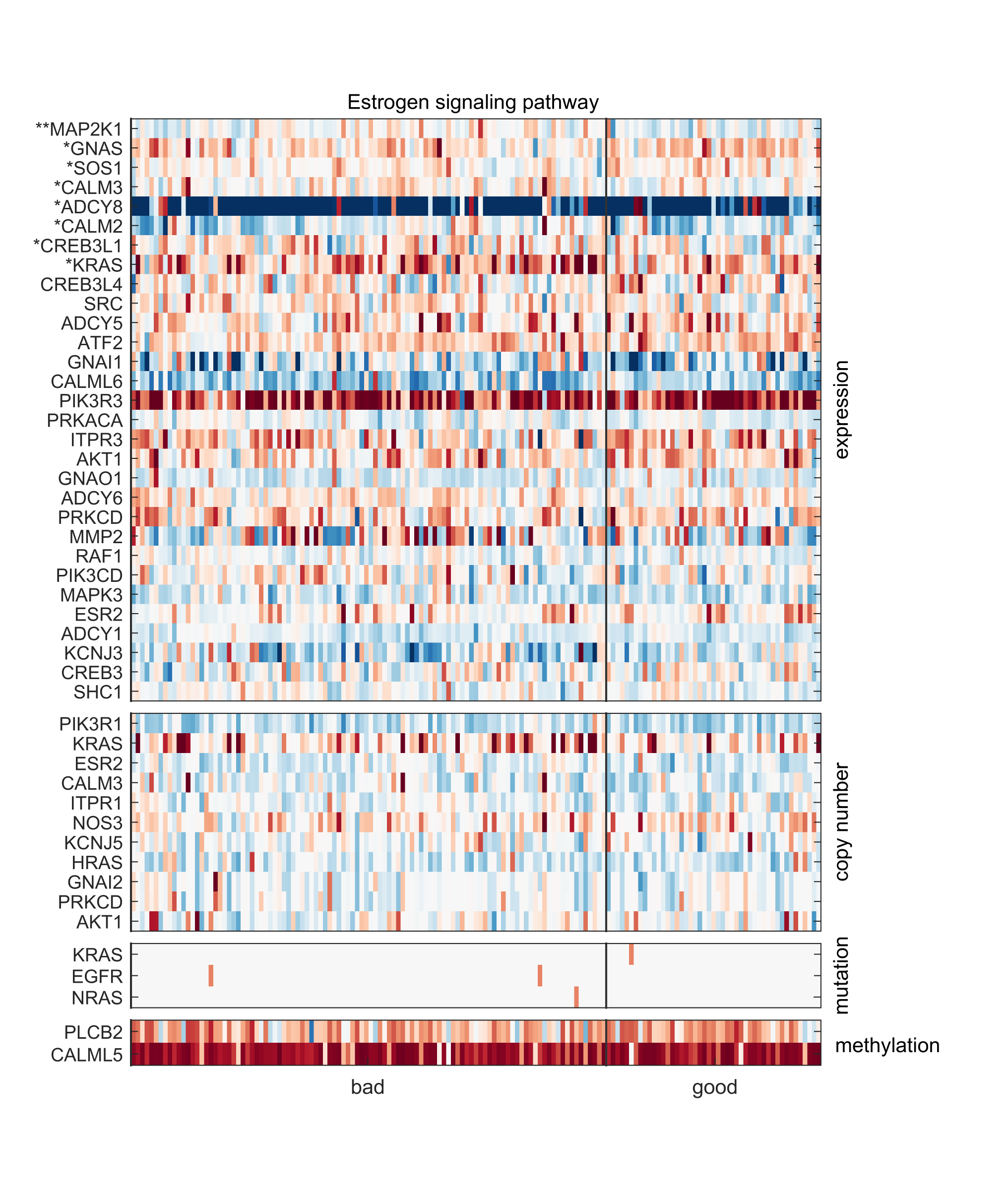

Supplement: S18 Fig — mRNA gene expression, mutation pattern, copy number status and methylation pattern for the genes of the Estrogen signaling KEGG pathway (hsa4915). Red = high value/presence, blue = low value. Methylation data are rescaled to the interval [0,1]. Genes are sorted according to the significance of a Kruskal-Wallis test, with the subtype as categorical factor (* = p<0.05, ** = p<0.01, *** = p<0.001). No FDR correction was applied. Maximum 30 genes per data type are shown. (TIF) [file pone.0133503.s018.tif]
