# Supplementary material for: Pathway Relevance Ranking for Tumor Samples through Network-Based Data Integration
Source: PLoS One. 2015 Jul 28;10(7):e0133503. doi: 10.1371/journal.pone.0133503 (PMC4517887; doi:10.1371/journal.pone.0133503)
Supplement: S2 Table — Pathway IDs correspond to KEGG identifiers. (PDF) [file pone.0133503.s020.pdf]

		Score		Rank	
**IDs**	**Names**	**SIM**	**FRQ**	**SIM**	**FRQ**
**4151**	PI3K-Akt signaling pathway	8.59	8.31	1	1
**4630**	Jak-STAT signaling pathway	8.31	5.41	2	5
**4152**	AMPK signaling pathway	8.22	7.57	3	2
**4350**	TGF-beta signaling pathway	7.39	5.38	4	6
**4012**	ErbB signaling pathway	7.26	5.04	5	12
**4550**	Signaling pathways regulating pluripotency of stem cells	7.12	5.20	6	10
**4921**	Oxytocin signaling pathway	7.08	5.36	7	7
**4510**	Focal adhesion	7.05	7.00	8	3
**4915**	Estrogen signaling pathway	6.80	4.13	9	30
**4917**	Prolactin signaling pathway	6.68	4.14	10	29
**4062**	Chemokine signaling pathway	6.48	4.38	11	21
**4024**	cAMP signaling pathway	6.38	4.13	12	31
**4014**	Ras signaling pathway	6.30	4.10	13	33
**4010**	MAPK signaling pathway	6.09	3.87	14	38
**4022**	cGMP-PKG signaling pathway	5.96	4.43	15	20
**4722**	Neurotrophin signaling pathway	5.95	5.07	16	11
**4930**	Type II diabetes mellitus	5.87	4.92	17	13
**4932**	Non-alcoholic fatty liver disease (NAFLD)	5.83	4.85	18	15
**4060**	Cytokine-cytokine receptor interaction	5.77	5.64	19	4
**4725**	Cholinergic synapse	5.76	3.15	20	58
**4919**	Thyroid hormone signaling pathway	5.73	4.36	21	22
**4015**	Rap1 signaling pathway	5.73	4.31	22	24
**4620**	Toll-like receptor signaling pathway	5.56	4.01	23	35
**4310**	Wnt signaling pathway	5.53	3.22	24	55
**4110**	Cell cycle	5.42	3.32	25	54
**4662**	B cell receptor signaling pathway	5.34	4.46	26	19
**4066**	HIF-1 signaling pathway	5.32	3.94	27	37
**830**	Retinol metabolism	5.30	5.36	28	9
**4150**	mTOR signaling pathway	5.10	4.60	29	18
**4611**	Platelet activation	5.03	3.55	30	46
**4920**	Adipocytokine signaling pathway	5.01	4.65	31	17
**3320**	PPAR signaling pathway	4.95	4.77	32	16
**4666**	Fc gamma R-mediated phagocytosis	4.92	4.21	33	27
**982**	Drug metabolism - cytochrome P450	4.81	4.90	34	14
**4390**	Hippo signaling pathway	4.78	4.11	35	32
**4610**	Complement and coagulation cascades	4.70	5.36	36	8
**4910**	Insulin signaling pathway	4.67	3.67	37	42
**350**	Tyrosine metabolism	4.61	4.27	38	26
**4660**	T cell receptor signaling pathway	4.60	3.47	39	50
**4261**	Adrenergic signaling in cardiomyocytes	4.58	3.48	40	49
**4916**	Melanogenesis	4.57	2.73	41	70
**4068**	FoxO signaling pathway	4.54	3.62	42	44
**4668**	TNF signaling pathway	4.52	3.83	43	40
**140**	Steroid hormone biosynthesis	4.51	4.17	44	28
**983**	Drug metabolism - other enzymes	4.49	3.87	45	39
**4380**	Osteoclast differentiation	4.44	3.62	46	45
**4512**	ECM-receptor interaction	4.35	4.33	47	23
**4750**	Inflammatory mediator regulation of TRP channels	4.29	3.15	48	59
**40**	Pentose and glucuronate interconversions	4.26	3.96	49	36
**4210**	Apoptosis	4.24	4.27	50	25
